# A Randomized, Open‐Label, Controlled Clinical Trial of Azvudine Tablets in the Treatment of Mild and Common COVID‐19, a Pilot Study

**DOI:** 10.1002/advs.202001435

**Published:** 2020-08-13

**Authors:** Zhigang Ren, Hong Luo, Zujiang Yu, Jingchao Song, Lan Liang, Ling Wang, Haiyu Wang, Guangying Cui, Yong Liu, Jin Wang, Qingquan Li, Zhaohai Zeng, Shengkun Yang, Guangzhong Pei, Yonghui Zhu, Wenbin Song, Wenquan Yu, Chuanjun Song, Lihong Dong, Chuansong Hu, Jinfa Du, Junbiao Chang

**Affiliations:** ^1^ Department of Infectious Diseases the First Affiliated Hospital of Zhengzhou University Zhengzhou 450052 China; ^2^ Guangshan County People's Hospital Guangshan County Xinyang 465450 China; ^3^ Department of Thoracic Surgery Henan Provincial Chest Hospital Zhengzhou 450008 China; ^4^ Medical Department Xixian people's Hospital Xixian Xinyang 464300 China; ^5^ Henan Key Laboratory of Organic Functional Molecule and Drug Innovation School of Chemistry and Chemical Engineering Henan Normal University Xinxiang 453007 China; ^6^ Department of Clinical Laboratory Henan Provincial Chest Hospital Zhengzhou 450008 China; ^7^ Henan Genuine Biotech Co., Ltd. 10 Fuxing Road, Xincheng District Pingdingshan Henan 467036 China; ^8^ Department of Orthopedics Huangchuan County People's Hospital Huangchuan County Xinyang 465150 China; ^9^ Henan new drug creation and drug safety evaluation Collaborative Innovation Center Zhengzhou University Zhengzhou 450001 China

**Keywords:** azvudine, COVID‐19, SARS‐CoV‐2

## Abstract

Coronavirus disease 2019 (COVID‐19) has spread worldwide. To date, no specific drug for COVID‐19 has been developed. Thus, this randomized, open‐label, controlled clinical trial (ChiCTR2000029853) was performed in China. A total of 20 mild and common COVID‐19 patients were enrolled and randomly assigned to receive azvudine and symptomatic treatment (FNC group), or standard antiviral and symptomatic treatment (control group). The mean times of the first nucleic acid negative conversion (NANC) of ten patients in the FNC group and ten patients in the control group are 2.60 (SD 0.97; range 1–4) d and 5.60 (SD 3.06; range 2–13) d, respectively (*p* = 0.008). The mean times of the first NANC of four newly diagnosed subjects in the FNC group and ten subjects in the control group are 2.50 (SD 1.00; range 2–4) d and 9.80 (SD 4.73; range 3–19) d, respectively (starting from the initial treatment) (*p* = 0.01). No adverse events occur in the FNC group, while three adverse events occur in the control group (*p* = 0.06). The preliminary results show that FNC treatment in the mild and common COVID‐19 may shorten the NANC time versus standard antiviral treatment. Therefore, clinical trials of FNC treating COVID‐19 with larger sample size are warranted.

## Graphical abstract

Azvudine treatment in the persistently mild and common COVID‐19 patients may shorten the nucleic acid negative conversion time versus standard antiviral treatment, regardless of whether the patients are newly diagnosed or have previously received routine treatment. Azvudine treatment could improve the lung function of patients. Moreover, the adverse events are not observed in patients receiving azvudine.

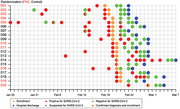

## 1 Introduction

In December 2019, severe acute respiratory syndrome coronavirus 2 (SARS‐CoV‐2) broke out in Wuhan, Hubei, China.^[^ ^1^ ^]^ As of 2020 July 1, more than 10 million patients have been confirmed, and over half of a million have died worldwide.^[^ ^2^ ^]^ Moreover, the number of confirmed and fatal cases continues to increase. However, at present, there is no specific drug for treating coronavirus disease 2019 (COVID‐19), and many critically ill patients have died without effective treatment. Thus, it is urgent to rapidly develop direct antiviral drugs to treat COVID‐19.

SARS‐CoV‐2 is a typically enveloped, single‐stranded positive RNA virus belonging to the Betacoronavirus genus, the Coronaviridae family and the Nidovirales order. RNA synthesis requires nucleosides and nucleotides as the raw materials.^[^ ^3^ ^]^ Therefore, in view of this characteristic, the nucleoside antiviral drugs most widely used in the clinic may have an anti‐ SARS‐CoV‐2 effect. Nucleoside antiviral drugs are synthetic and chemically modified nucleoside analogs that can enter host cells by imitating natural nucleosides. After entering the host cells, the nucleoside analogs are transformed into an active compound through the catalysis of kinase. The active compound was nucleoside triphosphate, which is embedded in viral DNA or RNA during viral DNA or RNA synthesis, leading to the termination of viral DNA or RNA chain synthesis and inhibition of viral replication.^[^ ^4^ ^]^ In addition, nucleoside antiviral drugs can also inhibit the activity of virus DNA‐dependent DNA polymerases (DdDps), RNA‐dependent DNA polymerases (RdDps) and RNA‐dependent RNA polymerases (RdRps), which can lead to the inhibition of viral replication.

Nucleoside antiviral drugs are characterized by a high antiviral efficacy and a high drug resistance barrier. Remdesivir, an unlisted nucleoside antiviral drug used abroad, has been tested in vitro to directly inhibit the activity of SARS‐CoV‐2^[^ ^5^ ^]^ and completed the phase III clinical trial for the COVID‐19 treatment. Although a recent report about the compassionate use of remdesivir for patients with severe COVID‐19 showed that clinical improvement was observed in 36 of 53 patients (68%),^[^ ^6^ ^]^ its phase III clinical trial result was unsatisfactory. However, at present, COVID‐19 is spreading rapidly around the world and has become a major threat to human health. Therefore, it is urgent to find more safe and effective nucleoside drugs against COVID‐19 as soon as possible.

Azvudine (FNC) is the first double‐target nucleoside drug that has demonstrated significant and broad‐spectrum in vitro antiviral effects against targets such as HIV,^[^ ^7^ ^]^ HCV,^[^ ^8^ ^]^ EV71,^[^ ^9^ ^]^ and HBV.^[^ ^10^ ^]^ Recently, azvudine showed potent antiviral activity against HCoV‐OC43 and SARS‐CoV‐2 in vitro (unpublished results). The result from phase II clinical trial (GQ‐FNC‐201) of treating HIV infection with azvudine showed that the drug had excellent efficacy and safety.^[^ ^11^ ^]^ Therefore, we speculate that azvudine has an anti‐COVID‐19 effect. Under the condition of COVID‐19 defined as public health emergency of international concern, we conducted this prospective, randomized, open‐label, controlled clinical trial to make a preliminary exploration of the efficacy and safety of azvudine tablets in the treatment of COVID‐19.^[^ ^12^ ^]^

## 2 Results

### 2.1 Patients

**Figure 1:**
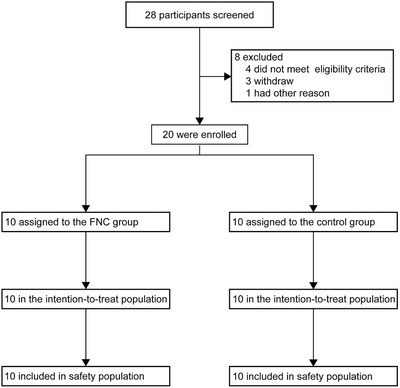
Trial profile.

From 2020 February 18 to 29, a total of 28 mild and common COVID‐19 patients were screened and only 20 mild and common COVID‐19 patients were eligible for enrollment in Guangshan County People's Hospital. Then patients were sequentially randomly assigned into the FNC group (*n* = 10) and the control group (*n* = 10) to receive therapy. All patients were cured and discharged before March 7 (**Figure** **1**).

The demographic and baseline characteristics of the patients were well matched between FNC group and control group at enrollment (**Table** **1**). The median age was 52 years (IQR 17–76), and 17 (85%) subjects had been exposed to confirmed patients or had returned from Wuhan. Most of the COVID‐19 patients were men (12 [60%]). A total of 17 patients had common COVID‐19, including eight patients in the FNC group (80%) and nine in the control group (90%). Both groups had one subject who underwent surgery (10%). A small number of patients had underlying diseases, including diabetes (1 [5%]) and cardiovascular disease (1 [5%]). The most common signs or symptoms at the onset of the illness were fever (11 [55%]), cough (10 [50%]), and fatigue (7 [35%]). The least common signs or symptoms were excessive phlegm (5 [25%]), dyspnea (3 [15%]), myalgia (2 [10%]), and headache (2 [10%]). None of the patients reported diarrhea at the onset of the illness. The mean time from illness to hospitalization was 6 d (IQR 1–28), and the mean total length of stay in the hospital was 11.5 d (IQR 6–32) (Table S1, Supporting Information).

**Table 1:** Demographics and baseline characteristics of the enrolled COVID‐19 patients on admission to hospital

	All patients (*n* = 20)	FNC group (*n* = 10)	Control group (*n* = 10)		*p* value
Characteristics					
Age [years]	52 (17–76)	52 (17–61)	50.5 (29–76)	0.515	
Sex	–	–	–	1.000	
Men	12 (60%)	6 (60%)	6 (60%)	–	
Women	8 (40%)	4 (40%)	4 (40%)	–	
Height [cm]	169 (154–178)	165 (154–172)	172 (154–178)	0.143	
Weight [kg]	71 (50–90)	64.5 (50–90)	75 (55–85)	0.303	
BMI [kg m^−2^]	25.325 (18.59–31.25)	24.545 (20.2–31.14)	25.325 (18.59–31.25)	0.533	
Clinical stage	–	–	–	0.531	
Mild	3 (15%)	2 (20%)	1 (10%)	–	
Common	17 (85%)	8 (80%)	9 (90%)	–	
Confirmed patient or Wuhan exposure	17 (85%)	8 (80%)	9 (90%)	0.531	
Surgery history	2 (10%)	1 (10%)	1 (10%)	1.000	
Current smoking	5 (25%)	3 (30%)	2 (20%)	0.606	
Any comorbidity					
Diabetes	1 (5%)	1 (10%)	0	0.305	
Hypertension	1 (5%)	0	1 (10%)	0.305	
Cardiovascular disease	1 (5%)	1 (10%)	0	0.305	
Signs and symptoms					
Fever	11 (55%)	4 (40%)	7 (70%)	0.178	
Highest temperature [°C]					
<37.3	9 (45%)	6 (60%)	3 (30%)	0.178	
37.3–38.0	4 (20%)	1 (10%)	3 (30%)	0.264	
38.1–39.0	7 (35%)	3 (30%)	4 (40%)	0.639	
Cough	10 (50%)	3 (30%)	7 (70%)	0.074	
Myalgia	2 (10%)	2 (20%)	0	0.136	
Fatigue	7 (35%)	5 (50%)	2 (20%)	0.16	
Excessive phlegm	5 (25%)	2 (20%)	3 (30%)	0.606	
Headache	2 (10%)	2(20%)	0	0.136	
Dyspnoea	3 (15%)	1 (10%)	2 (20%)	0.531	
Days from illness to hospitalization	6 (1–28)	4.5 (1–16)	7 (1–28)	0.453	
Days from confirmation to enrollment	4 (0–26)	3.5 (1–26)	4.5 (0–13)	0.567	
Systolic pressure, mm Hg	130 (108–160)	124.5 (117–144)	139 (108–160)	0.168	
Diastolic pressure, mm Hg	83 (65–110)	83.5 (74–96)	82 (65–110)	0.815	
Heart rate	84.5 (66–120)	88 (68–102)	79.5 (66–120)	0.727	
Respiratory rate	20 (18–24)	20 (18–24)	20 (19–24)	0.684	

Data are median (IQR) or *n* (%). *p* values comparing the FNC group and control group are from the Fisher's exact test, or Mann–Whitney U test. COVID‐19, coronavirus disease 2019; FNC, azvudine; BMI, Body mass index.

There was no difference in laboratory test results and computed tomography (CT) images during the screening between the FNC group and the control group. The mean blood cell counts were normal in the FNC group and control group, including white blood cells (4.84 × 10⁹ L^−1^ [IQR 2.94–8.71], FNC group; 6.185 × 10⁹ L^−1^ [3.03–8.18], control group), neutrophils (2.92 × 10⁹ L^−1^ [1.73–6.46], FNC group; 3.695 × 10⁹ L^−1^ [1.84–6.08], control group), and lymphocytes (1.565 × 10⁹ L^−1^ [0.89–2.83], FNC group; 1.58 × 10⁹ L^−1^ [0.99–2.65], control group). The mean prothrombin time was 12.5 s (7.7–17.8) in the FNC group and 13.15 s (9.6–14.2) in the control group. The mean aspartate aminotransferase and alanine transaminase levels were 23.7 U L^−1^ (10.3–76.8) and 36.8 U L^−1^ (11–181.3), respectively, in the FNC group and 23.5 U L^−1^ (14–40) and 28.3 U L^−1^ (10–111.9) in the control group (Table S2, Supporting Information). During the screening, all patients underwent chest CT images. Among them, four patients (20%) had single‐side lung invasion, and ten patients (50%) had bilateral lung invasion (**Table** **2**).

**Table 2:** Laboratory and CT images findings of COVID‐19 patients on enrollment

	All patients (*n* = 20)	FNC group (*n* = 10)	Control group (*n* = 10)	*p* value
Characteristics				
Red blood cell [10^12^ L^−1^]	4.43 (3.53–5.25)	4.4 (3.53–5.24)	4.66 (3.7–5.25)	0.512
Hematocrit [%]	41.05 (33.3–45.8)	40.75 (33.3–45.8)	41.7 (33.7–45.8)	0.444
Hemoglobin [g L^−1^]	136 (111–342)	139 (116–342)	132.5 (111–160)	0.684
Mean corpuscular volume [fL]	87.3 (83.1–102.1)	88.75 (85.7–94.3)	87.15 (83.1–102.1)	0.918
Mean erythrocyte hemoglobin [pg]	29.8 (26.2–33.1)	30.2 (29.3–33.1)	29.5 (26.2–31.2)	0.089
Mean erythrocyte hemoglobin content [g L^−1^]	337.5(295.2–358)	342 (330–351)	330 (295.2–358)	0.043
White blood cell [10^9^ L^−1^]	5.325 (2.94–8.71)	4.84 (2.94–8.71)	6.185 (3.03–8.18)	0.38
Neutrophil [10^9^ L^−1^]	3.38 (1.73–6.46)	2.92 (1.73–6.46)	3.695 (1.84–6.08)	0.467
Lymphocyte [10^9^ L^−1^]	1.57 (0.89–2.83)	1.565 (0.89–2.83)	1.58 (0.99–2.65)	0.812
Monocyte [10^9^ L^−1^]	0.34 (0.13–0.58)	0.29 (0.21–0.52)	0.34 (0.13–0.58)	0.579
Eosinophils [10^9^ L^−1^]	0.06 (0–0.25)	0.06 (0–0.19)	0.045 (0–0.25)	0.928
Basophilic [10^9^ L^−1^]	0.01 (0–0.3)	0.01 (0–0.3)	0.01 (0–0.03)	0.971
Platelet [10^9^ L^−1^]	191.5 (41–410)	160.5 (41–247)	218.5 (61–410)	0.101
Aspartate aminotransferase [U L^−1^]	23.7 (10.3–76.8)	23.7 (10.3–76.8)	23.5 (14–40)	0.579
Alanine aminotransferase [U L^−1^]	30 (10–181.3)	36.8 (11–181.3)	28.3 (10–111.9)	0.842
Cholinesterase [U L^−1^]	12 143 (6577–96 060)	36 778.5 (7763–93 460)	11 727.5 (6577–96 060)	0.878
Glutamyl transpeptidase [U L^−1^]	30 (11.4–181.1)	26 (11.4–181.1)	42 (14–114.3)	0.278
Total protein [g L^−1^]	65 (19.6–72.3)	64.5 (19.6–72.3)	65.3 (56.9–71.4)	0.842
Albumin [g L^−1^]	42.3 (32.8–52.1)	42.4 (33.8–52.1)	41. 65 (32.8–48)	0.397
Blood urea nitrogen [mmol L^−1^]	4.405 (3.38–6.12)	4.53 (3.42–5.86)	4.295 (3.38–6.12)	0.913
Uric acid [umol L^−1^]	285.5 (126.1–424)	295.3 (126.1–424)	277 (195–381.1)	0.996
Creatinine [umol L^−1^]	61.1 (42.1–93)	60.5 (42.1–93)	64. 3 (46.8–78)	0.734
Total bilirubin [umol L^−1^]	10.2 (6.2–19.6)	14.07 (7.6–19.6)	9.45 (6.2–18.7)	0.137
Direct bilirubin [umol L^−1^]	2.4 (1.1–9.36)	2.8 (1. 1––9.36)	2.15 (1.3–5.4)	0.182
Creatine kinase [U L^−1^]	74 (5–307.9)	75.25 (36.2–204)	74 (5–307.7)	0.78
Creatine kinase isoenzyme [U L^−1^]	9.26 (1.74–14.77)	9.75 (2.26–14)	9.1 (1.74–14.77)	0.456
Erythrocyte sedimentation rate [mm h^−1^]	17 (4–122)	9 (5–60)	30 (4–122)	0.351
C‐reactive protein [mg L^−1^]	4 (23.53%)	2 (25%)	2 (22.22%)	0.893
Procalcitonin [ng mL^−1^]	0.1 (0.04–0.5)	0.05 (0.04–0.12)	0.135 (0.04–0.5)	0.171
Thrombin time [s]	16.3 (13–28.6)	16.15 (13–28.6)	17.65 (13.3–24.2)	0.414
Activated partial thromboplastin time [s]	27.55 (20.2–35.2)	27.95 (20.2–33.3)	26.5 (22.1–35.2)	0.92
Prothrombin time [s]	12.75 (7.7–17.8)	12.5 (7.7–17.8)	13.15 (9.6–14.2)	0.9466
Fibrinogen [g L^−1^]	2.78 (1.13–4.61)	2.68 (1.65–4.61)	2.85 (1.13–3.73)	0.676
Oxygen saturation	0.98 (0.96–0.99)	0.98 (0.96–0.99)	0.98 (0.96–0.99)	0.856
Lung invasion	–	–	–	0.541
None	3 (15%)	2 (20%)	1 (10%)	–
Single side	4 (40%)	2 (20%)	2 (20%)	–
Both sides	10 (50%)	4 (40%)	6 (60%)	–

Data are median (IQR) or *n* (%). *p* values comparing the FNC group and control group are from the Fisher's exact test, or Mann–Whitney U test. COVID‐19, coronavirus disease 2019; FNC, azvudine.

Twelve patients (60%) had received routine treatment according to the “Diagnosis and treatment program trial version 5 guidelines” before enrollment. Among them, six treated subjects were in the control group (60%) and six were in the FNC group (60%). After enrollment, all patients received antiviral therapy. Two patients in the FNC group (20%) and six patients in the control group (60%) received antibiotic therapy. Three patients in the FNC group (30%) and four patients in the control group (40%) received traditional Chinese medicine. Five patients in the FNC group (50%) and ten patients in the control group (100%) received adjuvant medication. A total of three patients received oxygen support, two in the FNC group (20%) and one in the control group (10%) (**Table** **3**). Expect for FNC treatment and standard antiviral therapy, other treatments were symptomatic treatment according to the “Diagnosis and treatment program trial version 5 (or 6) guidelines,” and without additional treatments.

**Table 3:** Treatments of the enrolled COVID‐19 patients

	All patients (*n* = 20)		FNC group (*n* = 10)	Control group (*n* = 10)	*p* value
Treatment history	–		–	–	1.000
Treated patients	12 (60%)		6 (60%)	6 (60%)	
Naïve patients	8 (40%)		4 (40%)	4 (40%)	
Treatment					
Antiviral therapy	20 (100%)		10 (100%)	10 (100%)	1.000
Antibiotic therapy	8 (40%)		2 (20%)	6 (60%)	0.068
Chinese medicine	7 (35%)		3 (30%)	4 (40%)	0.639
Adjuvant medication	15 (75%)		5 (50%)	10 (100%)	0.01
Oxygen support		3 (15%)	2 (20%)	1 (10%)	0.531

Data are *n* (%). *p* values comparing the FNC group and control group are from Fisher's exact test. FNC: azvudine.

### 2.2 Rate of Nucleic Acid Negativity Conversion

**Figure 2:**
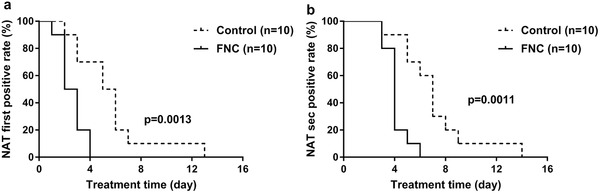
Kaplan–Meier curves of two consecutive nucleic acid testing negativity for a) the first and b) the second nucleic acid testing negativity in the FNC group and control group. Data are shown for ten patients assigned to FNC group and ten assigned to control group. Data are percentage (%). The differences between groups were using Log‐rank (Mantel‐Cox) test. On the fourth day after treatment, the rate of first negative conversion of nucleic acid achieved 100% in patients from the FNC group and 30% in patients from the control group. The Kaplan–Meier curves indicate the significant difference (*p* = 0.0013). On the sixth day, the rate of second nucleic acid negative conversion achieved 100% in patients from the FNC group and 40% in patients from the control group. The Kaplan–Meier curves indicate the significant difference (*p* = 0.0011). NAT, nucleic acid testing.

The rate of first negative conversion of nucleic acid after 4 d of treatment was 100% in the FNC group and only 30% in the control group (**Figure** **2**a). The Kaplan–Meier curves indicated the significant difference between two groups (*p* = 0.0013). The rate of nucleic acid negative conversion after 6 d of treatment was 100% in the FNC group and 40% in the control group (Figure 2b), with a significant difference (*p* = 0.0011).

### 2.3 Nucleic Acid Negative Conversion Time

**Figure 3:**
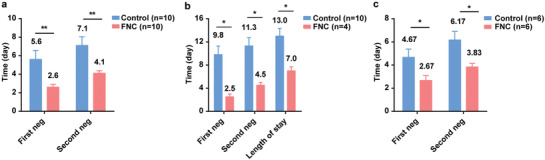
Comparison of the time of the first and the second nucleic acid testing negativity between a) all subjects in the FNC group and the control group (starting from the enrollment); b) four newly confirmed subjects in the FNC group and ten subjects in the control group (starting from the initial treatment); c) six treated subjects in the FNC group and six treated subjects in the control group (starting from the enrollment). Data are mean (SD). The differences between groups were analyzed using Student's *t*‐test. *p < 0.05, **p < 0.01, and ***p < 0.001. FNC, azvudine; first neg, first nucleic acid testing negativity; second neg, second nucleic acid testing negativity.

The mean times of the first nucleic acid negative conversion of the ten patients in the FNC group and the ten patients in the control group were 2.60 (SD 0.97; range 1–4) d and 5.60 (SD 3.06; range 2–13) d, respectively (difference, −3.00 d; 95% confidence interval [CI], −5.13 to 0.86; *p* = 0.008). The mean times of the confirmation of nucleic acid negative conversion were 4.10 (SD 0.88; range 3–6) d and 7.10 (SD 2.96; range 3–14) d, respectively (difference, −3.00 d; 95% CI, −5.05 to 0.95; *p* = 0.007) (**Figure** **3**a).

To eliminate the influence of grouping cut points on the treatment outcome, we analyzed the whole negative conversion time for the use of FNC alone. The mean times of the first nucleic acid negative conversion were 2.50 (SD 1.00; range 2–4) d for the 4 newly diagnosed patients in the FNC group and 9.80 (SD 4.73; range 3–19) d for 10 subjects in the control group (starting from the initial treatment) (difference, −7.30 d; 95% CI, −10.78 to −3.82; *p* = 0.01). The mean times of the confirmation of nucleic acid negativity conversion were 4.50 (SD 1.00; range 4–6) d and 11.30 (SD 4.50; range 5–20) d, respectively (difference, −6.80 d; 95% CI, −11.56 to −1.73; *p* = 0.01) (Figure 3b). The average lengths of stay in the hospital were 7.00 (SD 1.41; range 6–9) d and 13 (SD 4.30; range 7–22) d, respectively (difference, −6.0 d; 95% CI, −10.88 to −1.12; *p* =0.02).

Considering that six patients in each group had received standard antiviral treatment and symptomatic treatment before enrollment, we carried out a stratified analysis to understand the efficacy of FNC in treatment of previously treated COVID‐19 patients. The results are described as follows:

The mean times of the first nucleic acid negative conversion of the 6 treated patients in the FNC group and 6 treated patients in the control group were 2.67 (SD 1.03; range 1–4) d and 4.67 (SD 1.75; range 2–6) d, respectively (difference, −2.00 d; 95% CI, −3.85 to −0.15; *p* = 0.04). The mean times of confirmation of nucleic acid negative conversion were 3.83 (SD 0.75; range 3–5) d and 6.17 (SD 1.84; range 3–8) d, respectively (difference, −2.33 d; 95% CI, −4.14 to −0.53; *p* = 0.02) (Figure 3c). Moreover, the average times of treatment before enrollment in the FNC and control groups were 11.67 (SD 7.17; range 3–20) d and 7.00 (SD 3.63; range 2–13) d, respectively (difference, 4.67 d; 95% CI, −3.01 to 12.34; *p* = 0.20).

### 2.4 Improvement Rate of Chest CT Image

**Figure 4:**
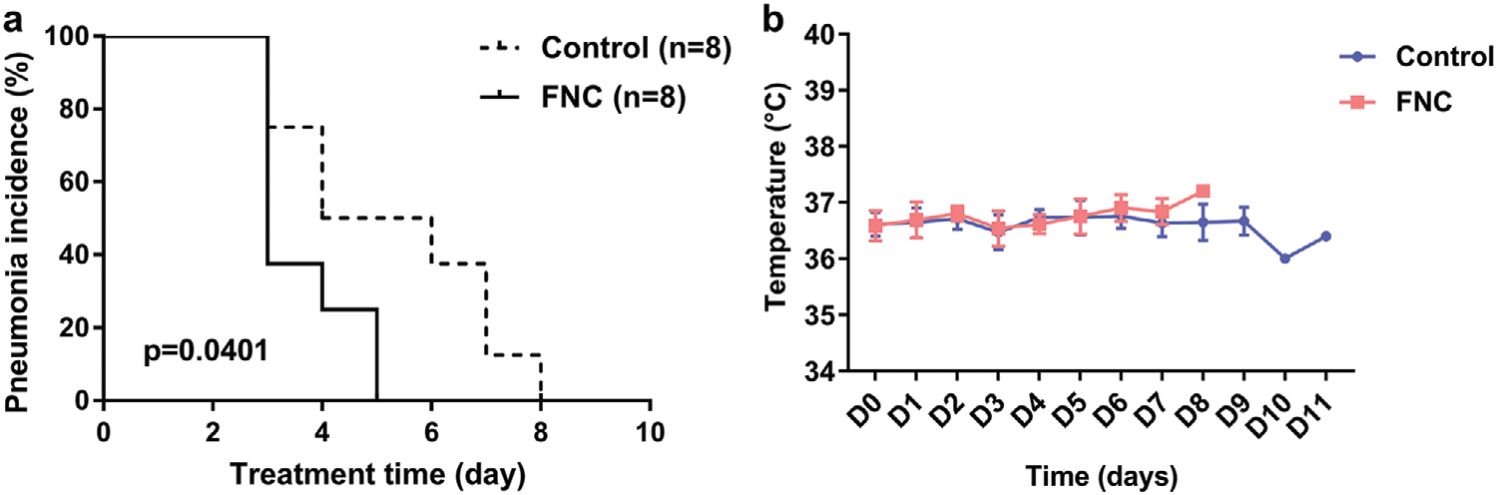
Secondary outcomes. a) Pneumonia incidence between eight subjects in the FNC group and eight subjects in the control group. Data are percentages (%). The differences between groups were analyzed using Log‐rank (Mantel–Cox) test. The pneumonia improvement time were shortened in the FNC group versus control group (*p* = 0.0401). b) The dynamic changes in body temperature of ten patients in the FNC and ten patients in the control group during the treatment. Data are mean (SD).

During the screening, the pulmonary CT of eight patients (001, 002, 003, 005, 011, 016, 019, 020) in the FNC group and eight patients (006, 007, 008, 012, 013, 014, 015, 018) in the control group showed abnormalities, including patchy blurred shadow, cord shadow, nodular shadow, or density shadow (Figure S2, Supporting Information). After enrollment and receiving FNC or routine treatment, the lungs in the subjects were improved (focus shrinking/absorption/no obvious abnormality). The pneumonia improvement rate was 100% after 5 d of treatment in the FNC group and 100% after 8 d of treatment in the control group. The pneumonia improvement time were shortened in the FNC group versus control group (*p* = 0.0401) (**Figure** **4**a).

During the screening, the pulmonary CT of two patients (004, 010) in the FNC group showed no obvious abnormalities. After enrollment, they received FNC treatment and maintained normal. The pulmonary CT images of two patients (009, 017) in the control group were missing.

### 2.5 Time of Body Temperature Returning to Normality

During the study, we monitored the body temperature of the participants every day. Due to the participants in the FNC and control groups receiving symptomatic treatment during the screening, their average body temperatures were basically normal. After enrollment and receiving FNC or routine treatment, the average body temperature remained normal, in the range of 35.60–37.40 °C (Figure 4b).

### 2.6 Improvement in Respiratory Symptoms and Signs

A total of ten subjects in both the FNC and control groups showed mild and occasional cough (slightly accompanied by pharyngeal discomfort or expectoration). After treatment, nine subjects returned to normality and remained normal until discharge. Only one subject in the control group (014) was transferred to another hospital to receive routine treatment on the ninth day after treatment due to epidemic management. The respiratory symptoms and signs of this subject had been improved, and his nucleic acid testing reached negativity on the 14th day.

### 2.7 Safety Evaluation and Adverse Events

**Figure 5:**
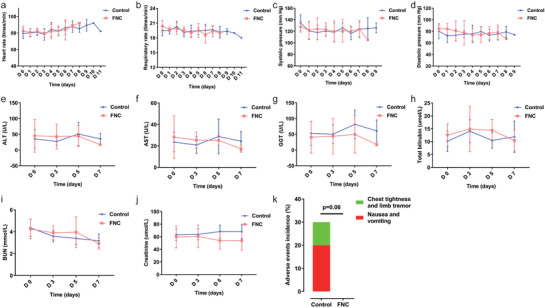
Safety and adverse events. During the treatment, the dynamic changes in a) heart rate, b) respiratory rate, c) systolic pressure, d) diastolic pressure, e) ALT, f) AST, g) GGT, h) total bilirubin, i) BUN, and j) creatinine of ten patients in the FNC group and ten patients in the control group. Data are mean (SD). ALT, alanine aminotransferase; AST, aspartate aminotransferase; GGT, glutamyl transpeptidase; BUN, blood urea nitrogen. k) Comparison of adverse events incidence of ten patients in the FNC group and ten patients in the control group. Data are percentages (%). The adverse event incidence was decreased in the FNC group (0%) versus the control group (30%) (*p* = 0.06).

The vital signs in the FNC group and control group, including heart rate, respiratory rate, systolic pressure, and diastolic pressure, were in the normal range during the treatment (**Figure** **5**a–d). The liver function in the FNC group and control group, including aspartate aminotransferase, alanine aminotransferase, glutamyl transpeptidase, and total bilirubin, were normal and did not significantly change during the treatment (Figure 5e–h). The kidney function in the FNC group and control group, including blood urea nitrogen and creatinine, were also normal during the treatment (Figure 5i,j).

During the study, no adverse events occurred in the FNC group, while three adverse events occurred in the control group, with an incidence of 30% (3/10) (*p* = 0.06) (Figure 5k). These adverse events were determined to be possibly related to the chloroquine phosphate treatment: one patient had anorexia, epigastric discomfort, nausea, abdominal distension without vomiting and abdominal pain; one patient developed chest tightness with limb tremor; and one patient had nausea and vomiting (stomach contents). The severity of the adverse events was grade 2 and disappeared after follow‐up. During the study, there were no serious adverse events, serious adverse reactions, important adverse events, deaths and so on.

## 3 Discussion

This pilot study is a randomized, open, controlled clinical trial to evaluate the efficacy and safety of azvudine tablets in the treatment of COVID‐19. Our trial found that azvudine may shorten the time of nucleic acid negativity conversion of persistent mild and common COVID‐19 patient versus standard antiviral therapy. Compared with a previous randomized clinical trial of using hydroxychloroquine in patients with mainly mild to moderate COVID‐19, our results is encouraging.^[^ ^14^ ^]^ The rate of nucleic acid negative conversion after FNC treatment is higher than hydroxychloroquine treatment (100% after 4 d treatment vs 73% after 28 d treatment). However, our sample size is smaller than hydroxychloroquine clinical trial, which may reduce the persuasion of our results.

We found that regardless of whether the patients were newly diagnosed or had previously received routine treatment, the time of nucleic acid negative conversion may be shortened with FNC versus standard antiviral therapy (4.1 d vs 7.1 d). Moreover, it was also found that although patients had received other treatment regimens, they did not affect the benefits of FNC treatment. In addition, FNC treatment can not only accelerate the elimination of the virus but also improve the lung function of patients, and maintain their vital signs.

Patients in our study are all persistently mild and common patients, with a median 6 d from illness to hospitalization, thus our results about the efficacy of FNC in treating COVID‐19 are applicable only to persistently mild and common patients. Although it is reported that COVID‐19 is basically a self‐limiting viral infection, and it resolves gradually over time, especially for mild and common cases, the most proportion of COVID‐19 patients,^[^ ^15^ ^]^ it does not mean that these patients do not need to receive treatment. On the contrary, these mild and common cases are the most proportion of COVID‐19, the major source of COVID‐19 transmission. These patients will recover faster after treatment compared with no medical treatment, and they just need fewer medical resources if receiving specific antiviral drugs timely. However, due to the lack of specific antiviral drug, the pandemic was not under the control and spread rapidly, which caused a large cumulative expense of medical resources. Fortunately, azvudine could reduce treatment time of mild and common patients and save a lot of medical resources. Therefore, azvudine may bring the hope for treating COVID‐19 and a larger controlled, randomized clinical trials are required to confirmed or contradict our findings.

The frequency of nucleic acid testing is not completely uniform for each subject, due to the difficulty of nucleic acid test during the pandemic. The testing time was determined by researchers based on the patient's condition and testing results. Moreover, all throat swab specimens were sent to the local Center for Disease Control (CDC) for nucleic acid testing by real‐time reverse transcriptase polymerase chain reaction (RT‐PCR). CDC determined whether to feed back the results on the day or the next day based on the received sample size and the submission time. These factors delayed the next sample collection. However, the first result of two consecutive nucleic acid negative conversion could reflect the efficacy of drugs. In addition, although the mean time from enrollment to the first nucleic acid testing was longer in the control group than that in the FNC group, it did not influence our results because the first testing result did not reach the real nucleic acid negativity conversion in patients who had longer testing interval time.

FNC in our study was given at a dose of 5 mg daily. The dose was determined according to the previous phase I and II clinical trials of FNC in the HIV treatment. In the phase I clinical trial, the climbing testings showed that 6 mg of FNC was still a safe dose for humans. Moreover, in the phase II clinical trial, no severe adverse event was associated with the FNC in the 2 mg group, 3 mg group, and 4 mg group. Given to the pandemic belonging to the public health emergency, we decided to use a dose with a better therapeutic effect and within a safe range. Thus, we used 5 mg FNC in this pilot study to make a preliminary exploration of the efficacy and safety of azvudine tablets in the treatment of COVID‐19. In our study, FNC treatment was well tolerated for patients. The vital signs, liver function and kidney function in both groups were normal. Moreover, three secondary adverse events were observed in the control group. It was confirmed that the adverse events were caused by chloroquine phosphate, which is consistent with other clinical trial data.^[^ ^16^ ^]^ There were no adverse events in the FNC group. Given to the short treatment time in FNC group, we will follow these patients to surveil and evaluate their prognosis.

Under the condition of COVID‐19 defined as public health emergency of international concern, we conducted this prospective, randomized, open‐label, controlled clinical trial to make a preliminary exploration of the efficacy and safety of azvudine tablets in the treatment of COVID‐19. It is inevitable that some disadvantages may exist in this study during the pandemic. First, the sample size of the groups were small, with only 20 people enrolled in this study owing to limited number of available patients. Our further recruitment was precluded by the rapid decline of new COVID‐19 cases due to successful restraint of COVID‐19 in early March 2020 in China, especially in Xinyang. However, our trial is a pilot study, in which sample size does not need to be calculated, aiming to make a preliminary exploration of the efficacy and safety. Second, the design of open‐label but not double‐blind, maybe influence the clinical decision‐making due to the knowledge of the treatment assignment. Third, there was no difference in demographic and baseline characteristics of the patients between two groups, but we did not test and compare the throat viral loads between two groups. Higher throat viral loads may raise more viral replication and delay the time of nucleic acid negativity conversion. Finally, the efficacy and safety of azvudine tablets in the treatment of severe and critically ill COVID‐19 was not acquired due to none of severe COVID‐19 patients admitted to hospital. Nevertheless, the results of randomized clinical trials of remdesivir^[^ ^17^ ^]^ and lopinavir–ritonavir^[^ ^18^ ^]^ in adults with severe COVID‐19 showed that no benefit was observed with remdesivir or lopinavir–ritonavir treatment. Therefore, trials with larger sample sizes are needed to confirm the efficacy and safety of FNC treatment for severe and critically ill COVID‐19 patients.

## 4 Conclusion

In summary, the present preliminary clinical trial results showed that the FNC treatment of mild and common COVID‐19 patients may shorten the time of nucleic acid negativity conversion versus standard antiviral treatment according to the Diagnosis and treatment program trial version 5 (or 6) guidelines. For newly diagnosed patients, the time of consecutive nucleic acid negativity conversion was shortened by an average of 4.5 d after treatment with FNC versus standard antiviral treatment. No drug‐related adverse effects were observed in patients treated with azvudine versus the 30% after treatment with standard antiviral drugs. The ongoing clinical trials are expected to verify the efficacy of FNC treatment in mild and common COVID‐19 and study whether FNC could benefit severe COVID‐19 patients (COVID‐19 = infection of SARS‐CoV‐2 = coronavirus disease).

## 5 Experimental Section

### Study Design

This prospective, randomized, open‐label, controlled clinical trial was performed at Guangshan County People's Hospital in China. This trial was approved by the institutional review board of the First Affiliated Hospital of Zhengzhou University (2020‐KY‐055) and Guangshan County People's Hospital (2020‐001) in accordance with the Good Clinical Practice guidelines of the International Conference on Harmonisation and the Declaration of Helsinki. The study was registered on chictr.org.cn with the number ChiCTR2000029853. All enrolled participants provided written informed consent.

### Patients

Patients meeting the following criteria were enrolled in the study: 1) age 18 and over, regardless of gender; 2) respiratory or blood samples that were tested positive for SARS‐CoV‐2 nucleic acid by RT‐PCR, or respiratory or blood samples that were tested highly homologous with the known SARS‐CoV‐2 by viral gene sequencing; 3) the confimation of COVID‐19 according to the diagnostic criteria of “the latest Clinical guidelines for novel coronavirus” issued by the World Health Organization (WHO) on 2020 January 28, and the diagnostic criteria from the “Diagnosis and treatment program trial version 5 (or 6) guidelines” issued by the National Health Commission of the People's Republic of China. All enrolled patients signed informed consent forms.

Exclusion criteria included 1) known or suspected allergy to the composition of azvudine tablets; 2) patients with malabsorption syndrome or any other condition that affects gastrointestinal absorption, the need for intravenous nutrition or an inability to take oral medication; 3) patients on anti‐HIV treatment; 4) patients with one of the following conditions: respiratory failure and the need for mechanical ventilation; shock; intensive care unit (ICU) monitoring and treatment for other organ failures; 5) pregnant women or those who were lactating or may have a birth plan during the trial period and within 6 months after the end of the trial; 6) patients participating in other clinical trials or using experimental drugs within 12 weeks before administration; and 7) patients with other conditions that were not suitable for participating in this experiment according to the judgment of the researcher.

The definition of mild COVID‐19 was patients with mild clinical symptoms and without signs of pneumonia in imaging; the definition of common COVID‐19 was patients with fever, respiratory, or other related symptoms, and with signs of pneumonia in imaging.

### Enrollment

The isolation ward was divided into two areas in hospital, one for suspected patients ward and the other for confirmed patients ward. Patients with fever, cough or other related symptoms, or clinical confirmed COVID‐19 were admitted to suspected patients ward. After hospitalization, the throat swab specimens were collected for nucleic acid testing by RT‐PCR the next day. Patients were transferred to confirmed patients ward if the nucleic acid testing was positive. Then the researcher evaluated the patient whether meeting the criteria and enrolled eligible patients after signing written informed consents. Patients with exposure to confirmed patients or Wuhan, but without symptoms, were isolated outside the hospital and collected throat swab specimens. Patients were admitted to hospital and transferred to confirmed patients ward if the result of nucleic acid testing was positive. Then researchers screened and enrolled eligible patients. A total of 28 mild and common COVID‐19 patients were screened and 20 patients were enrolled, without severe patients, because none of severe patients were admitted to hospital during the study.

### Randomization

Patients were randomly assigned in a 1:1 ratio to the FNC group or control group. Randomization was accomplished by using a random table that was generated in SAS software at 1:1. Each enrolled subject (meeting all the inclusion criteria and not meeting any of the exclusion criteria) was given a number, randomly assigned to the FNC group and control group according to a predetermined random table (Figure S1, Supporting Information), and received treatment according to the corresponding treatment regimen.

### Procedures

Patients in the FNC group were treated with oral azvudine tablets 5 mg d^−1^ (five tablets once a night) and symptomatic treatment. The dose of FNC was determined due to the results of phase I and II clinical trials of FNC in treatment with HIV. Patients in the control group were treated with standard antiviral treatment and symptomatic treatment. When patients developed clinical symptoms and signs, such as fever and cough, patients were treated with febrifuge or cough mixture, which was called symptomatic treatment. If not, patients were only treated with antiviral therapy. Standard antiviral treatment and symptomatic treatment were used according to the routine treatment of “Diagnosis and treatment program trial version 5 (or 6) guidelines” issued by the National Health Commission of the People's Republic of China.^[^ ^13^ ^]^ The standard antiviral drugs used in this trial were interferon alpha, kaletra, and ribavirin according to the Diagnosis and treatment program trial version 5 guidelines, and were interferon alpha, kaletra and ribavirin, chloroquine phosphate, and hydroxychloroquine sulfate according to the trial version 6 guidelines. On 2020 February 19, which overlapped with the study period, the “Diagnosis and treatment program trial version 5 guidelines” was updated to the sixth edition, and subsequent participants were given routine treatment according to the sixth edition treatment program. The drug was administered daily until the patient was discharged from the hospital or transferred to the corresponding department for the treatment of other diseases or death. During the treatment, no anti‐HIV drugs other than azvudine were used, and if there were patients with liver damage (the normal phenomenon of antiviral therapy), liver protection was provided in time. According to the Diagnosis and treatment program trial version 5 guidelines, the criteria for release and discharge were as follows: body temperature returned to normal for more than 3 d, respiratory symptoms showing obvious improvement, pulmonary imaging showing that the inflammation was obviously absorbed, and negative respiratory pathogenic nucleic acid testing for two consecutive assessments (with a sampling interval of at least 1 d). These criteria were not updated in the sixth edition.

The patient's vital signs, oxygen (via finger pulse oximetry), and respiratory symptoms and signs were monitored every day. On odd days and the discharge day, the patient's routine blood, erythrocyte sedimentation rate (ESR), C‐reactive protein, blood biochemistry, blood coagulation, myocardial markers, procalcitonin, myocardial zymogram, and arterial blood gas were monitored. The pneumonia change by chest CT images was monitored. Patients were taken CT scan once at the enrollment, every 3 d during the treatment, and at discharge. SARS‐CoV‐2 nucleic acids were tested by RT‐PCR after the patients began taking their drugs. The time of nucleic acid detection was determined by researchers based on the patient's condition and testing results.

### Outcomes

The primary outcomes were the rate of nucleic acid negativity conversion of SARS‐CoV‐2 and the negativity conversion time. Nucleic acid conversion rate was defined as the ratio of patients with negativity nucleic acid testing in FNC group to all patients in FNC group at a certain point in the follow‐up process. Secondary outcomes were the improvement rate of chest CT images, the time required for the body temperature returning to normal and the improvement in respiratory symptoms and signs. The improvement of chest CT images was defined as a significant reduction in the range of lesions and inflammation. The results for this outcome were double‐checked by two radiologists.

Safety was regularly assessed by monitoring vital signs (heart rate, respiratory rate, systolic pressure, diastolic pressure), changes in laboratory values (liver function, renal function), and adverse events (including type, incidence, severity, time and drug correlation, and assessment of severity according to the National Cancer Institute Common Terminology Criteria for Adverse Events [NCI CTCAE], version 5.0).

### Statistical Analysis

Continuous variables were presented with the form of mean (standard deviation) or median (interquartile range). Categorical variables were presented with the form of percentage. Differences between subjects in FNC group (*n* = 10, *n* = 4, or *n* = 6) and control group (*n* = 10 or *n* = 6) were analyzed by using Student's *t*‐test for normal continuous variables, Wilcoxon rank‐sum test for non‐normal continuous variables, and Fisher's exact test for categorical variables. Statistical analysis was performed using SAS software, version 9.4. Statistical significance was defined by *p* < 0.05 (two‐tailed), without post‐analysis and *α* adjustment. Data transformation, normalization, and evaluation of outliers were not used in this study.

## Conflict of Interest

The authors declare no conflict of interest.

## Author Contributions

Z.R., H.L., Z.Y., J.S., L.L., and L.W. contributed equally to this work. Study concept and design: J.C., Z.R., C.H., and J.D.; clinical trials: C.H., Z.R., H.L., J.S., L.L., Z.Y., L.W., Y.L., J.W., Q.L., Z.Z., S.Y., Y.Z., G.P., and W.S.; acquisition of data: Z.R., G.C., and Z.Y.; analysis and interpretation of data: J.C. and J.D.; research and development of Azvudine: J.C., J.D., W.Y., C.S., and L.D.; technical and material support: J.D. and Y.L.; drafting of the manuscript: Z.R., G.C., and H.W.; revision of the manuscript: J.C. and J.D.

## Supporting information

Supporting Information
