# Supplementary material for: A Randomized, Open‐Label, Controlled Clinical Trial of Azvudine Tablets in the Treatment of Mild and Common COVID‐19, a Pilot Study
Source: Adv Sci (Weinh). 2020 Aug 13;7(19):2001435. doi: 10.1002/advs.202001435 (PMC7404576; doi:10.1002/advs.202001435)
Supplement: Supplementary file 1 — Supporting Information [file ADVS-7-2001435-s001.pdf]

© 2020 Wiley-VCH GmbH

ADVANCED SCIENCE
Open Access

Supporting Information

for *Adv. Sci.,* DOI: 10.1002/advs.202001435

A Randomized, Open-Label, Controlled Clinical Trial of Azvudine Tablets in the Treatment of Mild and Common COVID-19, a Pilot Study

Zhigang Ren, Hong Luo, Zujiang Yu, Jingchao Song, Lan Liang, Ling Wang, Haiyu Wang, Guangying Cui, Yong Liu, Jin Wang, Qingquan Li, Zhaohai Zeng, Shengkun Yang, Guangzhong Pei, Yonghui Zhu, Wenbin Song, Wenquan Yu, Chuanjun Song, Lihong Dong, Chuansong Hu,* Jinfa Du,* and Junbiao Chang**

Supplementary Table S1. Clinical characteristics.

random number	001	002	003	004	005	006	007	008	009	010	011	012	013	014	015	016	017	018	019	020
groups	A	A	A	A	A	B	B	B	B	A	A	B	B	B	B	A	B	B	A	A
Nucleic acid detection	+	+	+	+	+	+	+	+	+	+	+	+	+	+	+	+	+	+	+	+
Treatment history (day)	3	20	16	naive	18	7	7	5	2	naive	naive	8	13	naive	naive	6	naive	naive	naive	7
clinical stage(C: common; M: mild)	C	C	C	M	C	C	C	C	C	M	C	C	C	C	C	C	C	M	C	C
Age (year)	58	56	51	24	44	47	54	47	58	17	48	46	56	39	29	61	58	76	57	53
Gender(F: female; M:male)	F	F	F	F	M	M	F	M	F	M	M	M	M	M	M	M	F	F	M	M
nationality	Han	Han	Han	Han	Han	Han	Han	Han	Han	Han	Han	Han	Han	Han	Han	Han	Han	Han	Han	Han
Height(cm)	165	156	160	154	170	173	156	175	172	165	168	175	170	160	178	164	172	154	172	172
Weight(kg)	55	62	60	50	90	80	58.4	72	55	70	67	80	85	80	75	57	75	60	80	75
BMI (kg/m2)	20.2	18.7	21.6	21.08	31.1	30.3	33.2	23.5	18.6	25.7	27.4	26.1	29.4	31.3	36.1	21.2	25.4	39	27	
Confirmed patient or Wuhan exposure	y	y	y	y	n	y	n	y	y	y	y	y	y	y	y	n	y	y	y	y
Current smoking	n	n	n	n	n	n	n	n	n	y	y	n	y	n	y	y	n	n	n	n
Any comorbidity																				
Diabetes	n	n	n	n	n	n	n	n	n	n	n	n	n	n	n	y	n	n	n	n
Hypertension	n	n	n	n	n	n	n	y	n	n	n	n	n	n	n	n	n	n	n	n
Cardiovascular disease	y	n	n	n	n	n	n	n	n	n	n	n	n	n	n	n	n	n	n	n
COPD	n	n	n	n	n	n	n	n	n	n	n	n	n	n	n	n	n	n	n	n
Malignancy	n	n	n	n	n	n	n	n	n	n	n	n	n	n	n	n	n	n	n	n
Chronic liver disease	n	n	n	n	n	n	n	n	n	n	n	n	n	n	n	n	n	n	n	n
Surgery history									y							y				
Signs and symptoms																				
Highest temperature, °C	36.8	36.8	38.1	36.2	39	36.6	38.2	37.3	37.6	36.5	37.4	38.5	38.3	37.4	38.1	39	36.7	37	36.5	37
Cough	n	y	Y	n	y	y	y	y	y	n	n	n	n	y	y	n	y	n	n	n
Myalgia	n	y	n	n	n	n	n	n	n	n	n	n	n	n	n	y	n	n	n	n
Fatigue	n	y	y	n	y	n	n	y	n	n	y	n	y	n	n	y	n	n	n	n
Excessive	n	n	n	n	y	n	n	n	y	n	n	n	n	n	Y	n	y	n	Y	n

phlegm																				
Headache	n	y	y	n	n	n	n	n	n	n	n	n	n	n	n	n	n	n	n	n
Diarrhoea	n	n	n	n	n	n	n	n	n	n	n	n	n	n	n	n	n	n	n	n
Dyspnoea	n	n	n	n	y	n	n	n	y	n	n	n	y	n	n	n	n	n	n	n
Days from illness to hospitalization	14	7	4	1	13	3	7	13	3	1	1	4	13	8	7	5	28	1	1	16
Total length of stay	9	25	24	7	32	15	11	12	12	9	6	16	22	15	7	11	8	12	6	11
Systolic pressure, mm Hg	120	124	124	144	117	108	136	160	146	125	123	147	142	130	116	135	151	126	136	131
Diastolic pressure, mm Hg	96	74	82	88	82	65	97	110	84	75	91	86	91	80	69	85	80	68	76	93
Heart rate	100	82	100	83	90	72	87	120	80	68	102	95	113	78	79	86	66	71	76	90
Respiratory rate	20	20	20	20	23	20	19	24	20	18	24	21	21	20	19	18	20	21	22	19

Supplementary Table S2: Laboratory findings on enrollment.

random number	001	002	003	004	005	010	011	016	019	020	006	007	008	009	012	013	014	015	017	018
groups	A	A	A	A	A	A	A	A	A	A	B	B	B	B	B	B	B	B	B	B
Red blood cell, RBC	4.16	3.53	3.93	4.37	4.36	5.24	4.43	4.79	4.8	4.58	4.9	3.7	4.89	4.97	5.25	3.88	4.32	5.12	4.43	4.25
Hematocrit	35.7	33.3	33.9	41	39.3	45.8	40.5	41.5	41.1	42	41.3	33.7	42.1	43.3	45.8	39.6	40.7	43.3	42.4	35.3
Hemoglobin	123	117	116	135	137	155	141	141	342	141	148	111	142	130	160	117	135	145	128	115
Mean corpuscular volume	85.8	94.3	86.3	94	90.1	87.4	91.3	86.6	85.7	91.7	84.3	91.1	86.1	87.1	87.2	102.1	94.3	84.5	95.7	83.1
Mean erythrocyte hemoglobin, MCH	29.6	33.1	29.5	31	31.4	29.5	31.7	29.4	29.3	30.8	30.2	30	29	26.2	30.5	30.2	31.2	28.2	28.9	27
mean corpuscular-hemoglobin concentration, MCHC	345	351	342	330	349	338	348	340	342	336	358	329	337	300.2	350	295.2	331	334	302.2	325
White blood cell, WBC	4.9	4.66	2.94	5.98	8.71	4.31	4.78	4.79	5.54	6.08	5.28	3.03	8.18	3.5	7.79	7	7.19	7.53	4.6	5.37
Neutrophil, N	2.49	2.92	1.73	2.79	6.46	2.14		3.38	3.69	4.52	3.55	1.84	6.08	2.22	5.87	4.92	3.84	4.84	2.12	3.28
Lymphocyte, L	2.13	1.32	0.93	2.83	1.63	1.72	1.81	0.89	1.5	1.14	1.38	1.05	1.71	0.99	1.32	1.51	2.65	2.06	2.18	1.65
Monocyte, M	0.21	0.29	0.27	0.27	0.4	0.26	0.52	0.49	0.29	0.37	0.34	0.13	0.34	0.31	0.58	0.43	0.42	0.44	0.2	0.34
Eosinophils, E	0.08	0.14	0.06	0.08	0.1	0.19	0.03	0.05	0.11	0.03	0.03	0.05	0.04		0.05	0.08	0.17	0.23	0.05	0.13
Basophilic, B	0.01	0.02	0.01	0.01	0.03	0.3	0	0	0	0.02	0.01	0	0.01	0.01	0	0.02	0.03	0.02	0.02	0.01
Platelet, PLT	247	211	122	162	190	209	133	119	159	41	193	170	410	134	294	267	61	227	236	210
Aspartate aminotransferase, AST	36.6	76.8	10.3	18		16	29.2	23.7	27	10.9	27.1	28.7	33.2	14	14	20	27	17	15	40
Alanine aminotransferase, ALT	36.8	181.3	11	13	80	15	44.3	20.6	38		36	26.6	111.9	10	16	50	40	18	18	30
Cholinesterase, CHE	61670	93460	66470	74690	10324	10486			7763	11887	####	96060	11056	84410		6577	10330	10592	78030	
Glutamyl transpeptidase, GGT	26	181.1	11.4	16	37	26	28.5	24.9	41		46.1	25.9	114.3	14	40	99	104	30	16	44

Total protein, TP	62.8	66.5	66	72.3	68.8	64.5	19.6	58.3	61.6		71.3	65	66.1	65.6	61.2	56.9	63.3	66.7	71.4	61.1
Albumin, ALB	40.5	42.2	40.3	48.3	52.1	50	42.4	33.8	43.1		48	39.5	42.3	38.2	39.8	32.8	43.8	47.5	41	43.2
Blood urea nitrogen, BUN	3.42	5.35	3.46	3.62	4.88	3.42	5.86	4.88	4.18	4.99	4.63	4.64	3.96	3.6	6.12	3.9	3.96	3.38	4.7	4.75
Uric acid, UA	202	424	189	205.9	392	352.9	303.6	126.1	386	287	374	195	362	284	200.2	270	381.1	370	235	195
Creatinine, CR	46.4	45.1	60.5	42.1	48.2	74.9	93	60.5	79.4	61.7	67	58.7	54.9	58	64.4	67.6	78	64.2	73	46.8
Total bilirubin, TB	7.6	15.1	14.5	19.6	9.81	10.9	14.07	18.13	8.5		9.5	6.2	12.8	6.3	18.7	10.2	9.4	14	8.5	6.4
Direct bilirubin, DB	1.1	9.2	2.8	3.4	2.23	2.4	5.06	9.36	2.1		2.1	1.5	2	2.2	5.4	3.1	1.9	2.8	2.6	1.3
Creatine kinase, CK	55.4	91.5	36.2	59	101	98	135.9	204	56	42	308	57	138.7	79	45	5		74	42	86
Creatine kinase isoenzyme, CK-MB	6.6	10	8.2	11.62	14	13.73	9.5	12	2.26	8.6	9.1	7.5	6.5	9.7	9.26	5.7		11.24	1.74	14.77
Erythrocyte sedimentation rate, ESR	44	6	60	9	14				6	5	14	61	30	38		122	5	4	60	20
C-reactive protein, CRP	N	N	Y	N	N	N	N	Y			N	N	N	Y	N	Y	N	N	N	
Procalcitonin, PCT		0.12		0.04		0.04	0.05			0.1	0.16	0.18	0.1	0.11	0.04	0.17			0.5	0.04
Prothrombin time, PT	17.8	7.7	11.3	14.6	9.8	14.6			12.8	12.2		10.8	9.6		14.1		13.6	14.2		12.7
Activated partial thromboplastin time, APTT	28.9	20.9	28.6	33.3	20.2	31.1			27.3	23.9		22.1	27.8		25.2		28.4	35.2		23.4
Fibrinogen, FIB	3.56	2.93	4.61	1.65	3.32	2.33			2.01	2.43		3.41	3.73		2.63		3.07	1.81		1.13
Thrombin time, TT	28.6	16.4	17.5	13	16.1	13.9			16	16.2		24.2	17.5		17.8		14.8	13.3		18.8
Oxygen saturation, SpO2	0.99	0.96	0.97	0.99	0.97	0.99	0.98	0.99	0.96	0.98	0.96	0.98	0.96	0.99	0.99	0.98	0.99	0.98	0.99	0.97

Supplementary Figure S1. Randomization and timeline.

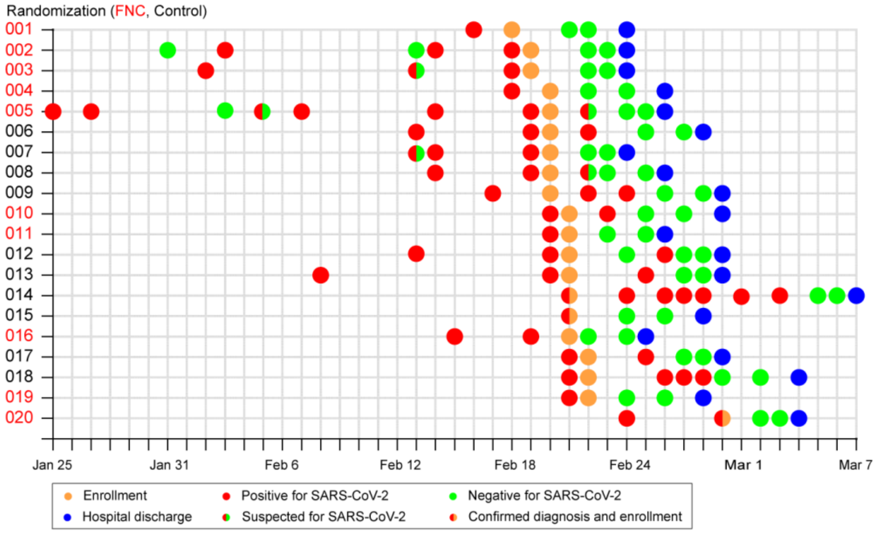

Twenty subjects were randomly assigned in a 1:1 ratio to the FNC group or control group according to a random table. The number was assigned according to the enrollment time. Red numbers indicate the FNC group, and black numbers indicate the control group. Red dots indicate dates on which RT-PCR testing was positive for SARS-CoV-2. Green dots indicate dates on which RT-PCR testing was negative for SARS-CoV-2. Orange dots indicate the enrollment dates. Blue dots indicate dates of discharge from the hospital. Red/green dots indicate dates of ambiguous RT-PCR test results. Red/orange dots indicate dates of confirmation time and enrollment time. The timeline includes the events of all subjects from confirmation to discharge from the hospital.

Supplementary Figure S2. Chest CT images.

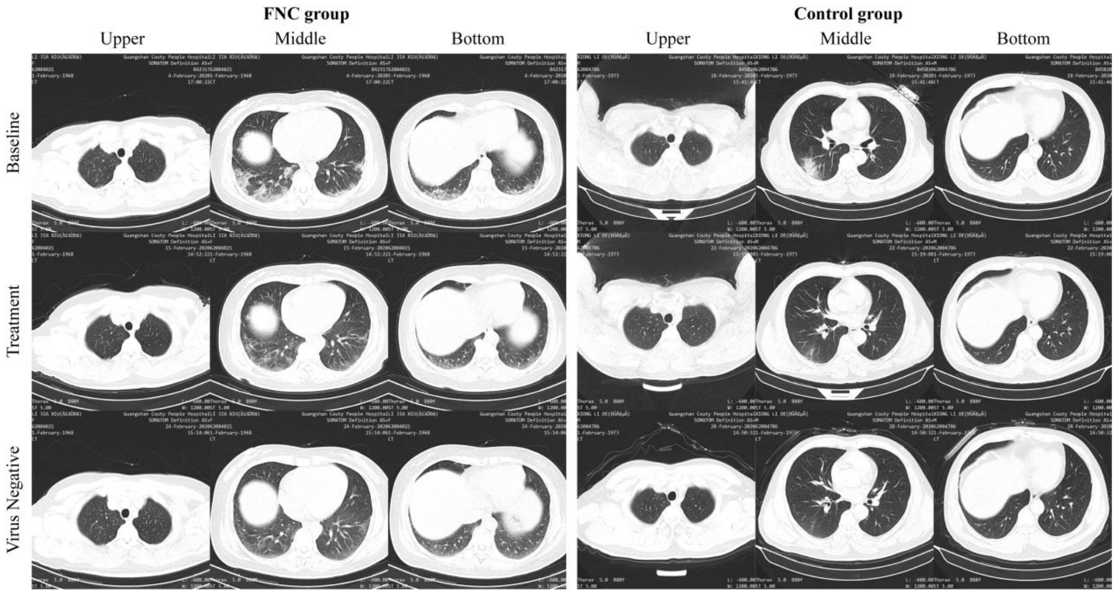

On the left side, the transverse chest CT images of subject 003 in the FNC group were showed from baseline, treatment to virus negativity, including the upper, middle, and bottom lungs. At baseline, chest CT images showed strip and flake high density shadow in both lungs, and small nodular high density shadow in left lung tongue. During the treatment, multiple patchy fuzzy shadows in both lungs and nodular shadow in the left lung tongue were observed. After nucleic acid negativity conversion, the scope of pulmonary lesions was reduced and the degree of inflammatory infiltration was significantly alleviated.

On the right side, the transverse chest CT images from subject 006 in the control group were showed from baseline, treatment to virus negativity, including the upper, middle, and bottom lungs. At baseline, chest CT images showed large areas of ground glass density shadow under the right lung pleura. During the treatment, patchy ground glass density shadow in right lung. After nucleic acid negativity conversion, the scope of pulmonary lesions was reduced and the degree of inflammatory infiltration was significantly alleviated.

Protocol

This trial protocol has been provided by the authors to give readers additional information about their work.

Protocol for: Ren Z, Luo H, Cui G, et al. A randomized, open-label, controlled clinical trial of Azvudine tablets in the treatment of Coronavirus disease 2019.

This document contains:

- The original protocol document (v1.0)

- The written informed consent for the enrolled participants. (v1.0)

- The random table that was generated by applying SAS at 1:1

阿兹夫定片治疗新型冠状病毒感染肺炎的随机、开放、对照临床试验

A randomized, open-label, controlled clinical trial of Azvudine tablets in the treatment of Coronavirus disease 2019

方案编号	FNC-Hope1
版本号	1.0
版本日期	2020 年 02 月 13 日
研究单位	光山县人民医院
主要研究者	裴广忠

保密声明

本试验方案属保密资料，用于提供给与本试验相关的医学专家、参加本试验的研究者等试验相关工作人员及承担本试验的医疗机构、伦理委员会和合同研究组织等相关业务委托机构。除向受试者说明情况之外，在未得到申办者事先书面同意的情况下，不得向第三方公开或泄漏本试验方案的任何内容。此外，本临床试验的部分或全部结果在向学会、杂志等外部发表时，需得到申办者的书面同意。

目录

缩略语表......1

试验方案摘要......2

1 研究背景......4

1.1 临床前药学相关资料......5

1.2 非临床研究......5

1.3 I 期临床试验总结......7

1.4 II 期临床试验总结......12

2 研究目的......16

3 研究设计......16

3.1 总体设计......16

3.2 随机方法和随机表......17

3.3 终止标准......17

4 受试者选择......17

4.1 入选标准......17

4.2 排除标准......18

4.3 退出标准......18

5 受试者的治疗......19

5.1 研究药物......19

5.2 治疗方案......19

5.3 合并用药......19

6 试验步骤......20

6.1 有效性及安全性观察指标......20

6.2 有效性及安全性观察时点......21

6.3 不良事件的随访......22

7 终点指标......22

7.1 疗效指标 22

7.2 安全性指标 22

8 安全性评价 23

8.1 安全性评价参数 23

8.2 安全性评估 23

8.3 不良事件 23

8.4 妊娠 27

9 数据管理和统计分析 27

9.1 数据管理 27

9.2 统计分析 28

10 研究管理 29

10.1 伦理委员会 29

10.2 知情同意 29

10.3 资料的保存 29

10.4 研究认可 29

11 参考文献 29

缩略语表

缩写语	中文名	缩写语	中文名
AE	不良事件	LYMPH#	淋巴细胞绝对数
ALP	碱性磷酸酶	MCV	平均红细胞体积
ALT	丙氨酸氨基转移酶	MCH	平均红细胞血红蛋白含
Alb	白蛋白	MCHC	平均红细胞血红蛋白浓
APTT	活化部分凝血活酶时间	MRT	平均驻留时间
AST	天门冬氨酸氨基转移酶	MedDRA	国际医学用语词典
AUC	药-时曲线下面积	MONO#	单核细胞绝对数
BASO#	嗜碱性粒细胞绝对数	NEUT#	嗜中性粒细胞绝对数
CDE	药品审评中心	NOAEL	未见明显损害的剂量
CI	可置信区间	PBMCs	外周单核细胞
CK	肌酸激酶	PLT	血小板计数
CK-MB	磷酸肌酸激酶同工酶	PPS	符合方案集
CL	清除率	PT	MedDRA 编码的首选语
C_{max}	血药峰浓度	PV	药物警戒
CRF	病例报告表	PT	凝血酶原时间
CRO	合同研究组织	RBC	红细胞计数
DBIL	直接胆红素	RNA	核糖核酸
DNA	脱氧核糖核酸	RT	逆转录酶
EC_{50}	半最大效应浓度	RT-PCR	逆转录-聚合酶链反应
EC_{50}	半最大效应浓度	SAE	严重不良事件
EFV	依非韦伦片	Scr	血肌酐
EOS#	嗜酸性粒细胞绝对数	SD	标准差
FAS	全分析集	SOC	系统器官分类
FIB	纤维蛋白原	SS	安全性数据集
FNC-TP	FNC 的三磷酸酯	SUSAR	非预期严重不良反应
FNC	阿兹夫定片	TAF	替诺福韦二代
3TC	拉米夫定片	TI	治疗指数
GCP	药物临床试验质量管理规范	T_{max}	血药浓度达峰时间
Glu	血糖	TP	总蛋白
GGT	谷氨酰基转移酶	TDF	富马酸替诺福韦二吡呋
HAART	高效抗逆转录病毒治疗	TBIL	总胆红素
HCV	丙型肝炎病毒	TT	凝血酶时间
HIV	人类免疫缺陷病毒	$t_{1/2}$	半衰期
HBsAg	乙肝表面抗原	UA	尿酸
Hb	血红蛋白	ULN	正常上限
HCT	红细胞比积	UREA	尿素
IB	研究者手册	Vd	分布容积
ICH	人用药品注册技术要求国际协	WBC	白细胞计数
Ig	单次灌胃	WHO	世界卫生组织
iv	静脉注射	2019-nCoV	新型冠状病毒

试验方案摘要

**研究题目**	阿兹夫定片治疗新型冠状病毒感染肺炎的随机、开放、对照临床试验
**药物名称**	阿兹夫定片
**研究目的**	在临床常规治疗的基础上，评价阿兹夫定片治疗新型冠状病毒感染肺炎的有效性和安全性
**研究设计**	随机、开放、对照临床试验
**受试人群**	新型冠状病毒感染的肺炎患者
**样本量**	计划入组 20 例受试者，每组 10 例。
**病例选择**	**1、入选标准：**<br>受试者应全部符合以下标准才能进入试验：<br>（1） 年龄≥18 周岁（含临界值），性别不限；<br>（2） 呼吸道标本或血液标本实时荧光 RT-PCR 检测 2019-nCoV 核酸阳性，或者，呼吸道标本或血液标本病毒基因测序，与已知的 2019-nCoV 高度同源；<br>（3） 确诊为新型冠状病毒感染者，患者符合世界卫生组织（WHO）在 2020 年 1 月 28 日发布的《最新版针对新型冠状病毒的临床指南》和中国卫健委《关于印发新型冠状病毒感染的肺炎诊疗方案（试行第五版）》的诊断标准；<br>（4） 已签署知情同意书。<br>**2、排除标准：**<br>受试者符合以下任何一项标准将不得进入本研究：<br>（1） 已知或怀疑对阿兹夫定片的组成成分过敏；<br>（2） 合并有吸收障碍综合征，或其它任何对胃肠道吸收产生影响的状

	况，需要静脉营养或不能口服药物的患者；<br>（3） 正在接受抗 HIV 病毒治疗的患者；<br>（4） 患者出现以下情况之一者：出现呼吸衰竭，且需要机械通气；出现休克；合并其他器官功能衰竭需 ICU 监护治疗；<br>（5） 妊娠期、哺乳期妇女或在试验期间及结束 6 个月内有生育计划；<br>（6） 给药前 12 周内参加过其它临床试验或者正在使用试验性药物；<br>（7） 根据研究者的判断，具有不适合参加本试验的其它情况。
**研究药物**	名称：阿兹夫定片（FNC）<br>规格：1mg/片<br>来源：河南真实生物科技有限公司<br>贮存条件：密封、室温保存
**治疗方案**	试验组：阿兹夫定片+常规治疗<br>对照组：常规治疗<br>阿兹夫定片：5片/次，1次/日，口服。<br>常规治疗：参照中国卫健委《关于印发新型冠状病毒感染的肺炎诊疗方案（试行第五版）》，给予受试者相应的治疗措施。<br>疗程：每日给药直至受试者解除隔离出院或根据病情转至相应科室治疗其他疾病或病死。
**合并治疗**	**禁止使用的治疗：**<br>试验期间，受试者不得使用其它抗HIV病毒药物，以及研究者禁止使用的其它药物。<br>**允许使用的治疗：**<br>抗病毒治疗过程中，肝损害是常见的不良反应。试验期间，如果发生肝损害，需要及时给与保肝治疗。

疗效指标	（1） 体温恢复正常的时间和比例；<br>（2） 呼吸道症状及体征（肺部啰音、咳嗽、咯痰、咽痛等）改善的时间和比例；<br>（3） 腹泻、肌痛、乏力等其他症状改善的时间和比例；<br>（4） 肺部影像学改善的时间和比例；<br>（5） 2019-nCoV 核酸转阴的时间和比例；<br>（6） 氧合指数改善的时间和比例；<br>（7） CD4 计数改善的时间和比例；<br>（8） 轻型/普通型转重型比例，重型转危重型比例；<br>（9） 住院时间；<br>（10） 死亡率。
安全性指标	✓ 生命体征（体温、心率、呼吸、血压）；<br>✓ 血常规、血沉、白介素 6、C-反应蛋白、肝功能、肾功能、凝血功能、血乳酸、心肌酶谱、心肌标志物、降钙素原、妊娠试验；<br>✓ 心电图；<br>✓ 不良事件：包括类型、发生率、严重程度、时间和药物相关性，严重程度参考 CTCAE（5.0 版）进行评估。

阿兹夫定片治疗新型冠状病毒感染肺炎的随机、开放、对照临床试验方案

1 研究背景

2019 新型冠状病毒（2019-novel coronavirus，2019-nCoV）属于新发现的β属冠状病毒。2019-nCoV 感染肺炎疫情已是国际关注的突发公共卫生事件。新型冠状病毒感染肺炎可引起人呼吸道症状、发热、咳嗽、气促和呼吸困难等，在危重病例中，病毒主要导致严重的全身炎症反应（细胞因子风暴），引起急性肺损伤、重症肺炎、急性呼吸窘迫综合征的（ARDS）、严重低氧血症、感染性休克，肾衰竭，甚至死亡，目前该病毒已表现出强的传染性。自去年 12 月湖北省武汉市陆续发现了多例新型冠状病毒感染的肺炎患者，随着疫情的蔓延，截止 2020 年 02 月 12 日止，全国已累计 44763 例确诊病例，其中重症病例 8204 例，累计死亡病例 1115 例。目前，新型冠状病毒感染在全球，尤其在中国的扩展迅速，成为人类健康的重大威胁。

冠状病毒的复制生命周期中的不可缺少的一个环节是病毒的 RNA 的合成。通常病毒 RNA 的复制利用天然核苷为底物完成。但是，快速复制的病毒 RNA 对底物的选择性显著降低，极其容易错误地将人工合成的核苷结合进入病毒 RNA，使其病毒 RNA 链的合成终止，达到杀死病毒的结果。

核苷在抗病毒感染的治疗中起着至关重要的作用。如抗艾滋病药中有 7 款核苷药，如 3TC、FTC、TDF 或 TAF，全球每年每天有约 3000 万病人服用核苷类抗艾滋病药。最重要、应用最广泛的乙肝药包括 TAF、TDF、Entecuvir 都是核苷类药物。丙肝药中的索非布韦更是丙肝治愈中的革命性的药物，使丙肝的治愈率达到 95%以上。

可见，核苷类药物在抗病毒治疗中有着不可替代的地位。

核苷类抗病毒药物有抗病毒疗效高，耐药屏障高的特点。2019-nCoV 冠状病毒是新型 RNA 病毒，有几个核苷被报道显示体外抗冠状病毒效果，如 6-azauridine，carbavir, 尤其是 Remdesivir，体外显示较强的抗冠状病毒的作用。这些核苷药物都在早期研发阶段，6-azauridine 的毒性较大，选择性较低，Carbovir 的抗病毒活性较低。Remdesivir 的临床安全性还有待探索。因此，寻找新型核苷类抗冠状病毒药物十分必要。

I 类新药阿兹夫定（Azvudine，FNC）是新型核苷类逆转录酶抑制剂，据 Roche（罗氏）报道，体外试验证明，阿兹夫定能显著地抑制 RNA 病毒，如丙肝病毒（IC50=24nM, JBC, 2008, 283, 2167）、手足口病毒（RNA）、非洲猪瘟病毒（RNA）及乙肝病毒（DNA 病毒 EJMC 2015,101,103），显著抑制 HIV（RNA 病毒），并且已经完成抗 HIV 二期临床试验（GQ-FNC-201），人体有效性和安全性均良好。以上数据证明阿兹夫定具有显著而广谱的抑制 RNA 和 DNA 病毒的作用，并且临床安全性良好。因此，阿兹夫定很可能具有抗冠状病毒（2019-nCoV）的作用。面对这样 2019-nCoV 病毒引起的肺炎疫情严峻的问题，研制安全有效的抗 2019-nCoV 药物乃当务之急，建议开展临床试验探索阿兹夫定抑制冠状病毒的疗效。

1.1 临床前药学相关资料

化学名称：4’-叠氮-2’-脱氧-2’-氟阿拉伯胞苷，1-(4-叠氮-2-脱氧-2-氟-beta-D-阿拉伯核糖)胞嘧啶，简称 FNC。

化学结构式：

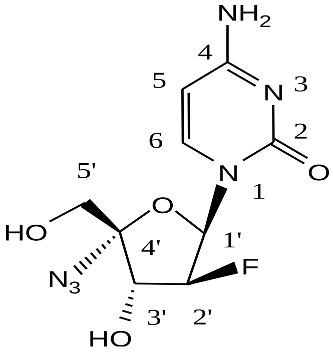

分子式：$C_9H_{11}FN_6O_4$

分子量：286.220

性状：无臭、无味的淡黄色晶体。

1.2 非临床研究

1.2.1 药理作用

FNC 是世界首个艾滋病毒逆转录酶（RT）与辅助蛋白 Vif 双靶点抑制剂药物，能够选择性进入 HIV 靶细胞 PBMCs 中的 $CD4^+T$、$CD14^+T$ 细胞，发挥抑制病毒复制功能。FNC 的三磷酸酯（FNC-TP）可以有效抑制重组 HIV 逆转录酶活性。

FNC 与拉米夫定（3TC）联合应用时，有协同/相加作用。

在抗 HIV-1 活性试验中，FNC 对实验株、耐药株和临床分离株有很好的抑制效果，EC_{50} 在 pM 水平，明显低于 3TC，治疗指数（TI）高于或相当于 3TC。

体外试验结果表明，FNC 对于 HIV-1 实验株敏感的人 T 淋巴细胞传代株毒性低。

1.2.2 动物毒理学研究

犬给予 FNC 39 周毒性试验结果表明，0.3mg/kg 高剂量水平下，主要毒性靶器官为胃肠道及免疫造血系统，停药 4 周后，毒性反应可基本恢复。0.03、0.1mg/kg 剂量组未见毒性反应，以上无毒性反应剂量（NOAEL）的系统药物暴露量相当于 II 期临床最高使用剂量（4mg）人体暴露量的 20-30 倍。

与其它许多核苷类似物一样，FNC 在遗传毒性试验中有部分剂量呈阳性。

在 Ames 试验中，未引起全部测试菌株基因突变的最高剂量为 200μg/皿，相当于 II 期临床最高使用剂量人体暴露量的 50000 倍。

在体外 CHL 细胞染色体畸变试验中，未引起 CHL 细胞染色体结构畸变率升高的剂量为 7.5μg/mL，相当于 II 期临床最高使用剂量人体暴露量的 1875 倍。

但犬给予 FNC 39 周毒性试验中，在相当于或高于 II 期临床最高使用剂量的给药剂量（0.03、0.1、0.3mg/kg）下，均未见遗传相关毒性反应。

FNC 对雄性小鼠的交配或生育力以及精子和后代均无影响。

雌鼠给予 1.5mg/kg FNC，生育力及胚胎发育未出现异常；孕鼠给予 0.5mg/kg FNC，孕鼠、胚胎及胎仔发育未见毒性反应；围产期孕鼠未发现毒性反应的剂量为 0.5mg/kg，对围产期孕鼠给予 1.5mg/kg FNC，母鼠见轻微毒性，子代大鼠未见明显异常。以上无毒性反应剂量相当于 II 期临床最高使用剂量的 1-3 倍。

FNC 仅可少量通过血乳屏障，经乳汁排出。

FNC 对亲代母兔、胚胎及胎仔发育的 NOAEL 均为 1.0mg/kg，相当于 II 期临床最高使用剂量的 5 倍。

上述研究中，大鼠和兔的系统药物暴露量是人体暴露量（II 期临床最高使用剂量）的 50-100 倍。

1.2.3 药代动力学

吸收：

单次灌胃（ig）给予犬、大鼠低、中、高三个不同剂量药物，C_{max}和 AUC_{0-t}均随剂量增加而增加，半衰期基本不变，符合线性动力学。多次给药结果表明首次和末次 C_{max}和 AUC_{0-t}差异无统计学意义，即 C_{max}和 AUC_{0-t}无累积。

雌性大鼠 ig 和静脉注射（iv）给予 FNC 0.52mg/kg 后，AUC_{0-t} 分别为（2754.94±166.93）ng·h/mL 和（3339.89±1295.10）ng·h/mL，绝对生物利用度为 82.49%；雄性大鼠 ig 和 iv 给予 FNC 0.52mg/kg 后，AUC_{0-t} 分别为（1562.86±417.23）ng·h/mL 和（1733.85±126.03）ng·h/mL，绝对生物利用度为 90.14%。

分布：

FNC 主要分布在免疫器官胸腺和脾脏，且消除较慢。胸腺中 FNC 含量在给药后 12h 达到最大，24h 已明显下降，72h 浓度很小，基本消除。脾脏中 FNC 的浓度在给药后 7h 达到最大，48h 已明显下降。在心、肝等组织分布较少且消除较快，在脂肪、脑和脊髓组织中含量最少。

FNC 与血浆蛋白结合程度很低，在血浆中主要以游离形式存在。

代谢和清除：

FNC 主要以原型及代谢物形式通过肾脏排泄。

1.3 I 期临床试验总结

1.3.1 单次给药安全性、耐受性及药代动力学试验总结

本研究共入组 40 例未接受过治疗的 HIV 感染者进行单次给药，剂量组和各组例数见表 1：

表 1 单次给药总体情况

试验组别	1	2	3	4	5
给药剂量	1mg	2mg	3mg	4mg	5mg
受试例数	2+6	8	8	8	8

本次研究中未出现严重不良反应，不良反应主要为发热、头痛、头晕、恶心、呕吐、腹泻，严重程度均为 1 级，除了 2 例受试者发热采用扑热息痛（1 片/次，2

次，1 天）和泰诺（1 片）治疗外，均未采取任何措施自行恢复。

阿兹夫定片口服给药后，体内血浆药物浓度较低，且个体差异较大。1mg 剂量组未检测出血药浓度，2mg 剂量组中 2 例受试者未检测出血药浓度，故未进行药代参数分析。3mg、4mg、5mg 组 AUC_{0-t} 逐渐增大，且 4mg 与 3mg 组有统计学差异。4mg 剂量组 C_{max} 均值最高，与 3mg 和 5mg 并无统计学差异。各剂量组 T_{max}、MRT_{0-t} 及 $t_{1/2}$ 相近，且无统计学差异。T_{max} 均在 1h 左右，MRT_{0-t} 及 $t_{1/2}$ 均大于 4h 和 8h，提示 FNC 在体内吸收较快，消除较慢。

受试者排尿量差异较大。同剂量组阿兹夫定排泄量个体差异较大。各剂量组阿兹夫定平均排泄率相近，无统计学差异。而 0-12h 排泄量占总排泄量 0-24h 的 70%以上。随着给药剂量增加，尿中阿兹夫定总排泄量增加，相关系数为 0.903。

1.3.2 多次给药安全性、耐受性及药代动力学试验总结

本研究共入组 16 例未接受治疗的 HIV 感染者进行多次给药，剂量组、各组例数及给药方法见表 2：

表 2 多次给药总体情况

试验组别	A	B
给药剂量	2mg	4mg
受试例数	8	8
给药方法	2 次/天，2 片/次，两次给药间隔 12 小时，连续口服 7 天	每天早晨空腹给药 1 次，4 片/次，连续口服 7 天

2mg和4mg组分别有3例和2例受试者发生5次和2次AE，仅4mg组1例1次AE判定为与研究药物可能有关，严重程度II级，表现为给药第4天发生中性粒细胞绝对值降低，未给与治疗，出组检查时已恢复至消失/复常，其余6次AE严重程度均为I级，5次AE

判定为与研究药物可能无关，1次AE肯定无关。

2mg 组首末次给药后的 C_{max}、AUC_{0-12}、T_{max}、$t_{1/2}$ 分别为：1.95±1.1、2.17±0.7（ng·mL^{-1}）；6.95±2.4、14.79±3.6（ng·h·mL^{-1}）；1.19±1.3、1.94±2.5（h）； 7.43±2.6、9.69±2.2（h）。末次 $AUC_{(0-12h)}$/首次 $AUC_{(0-12h)}$比值为 249.55±159.78% ，说明 HIV 感染者体内阿兹夫定有一定蓄积。

4mg 组首末次给药后的 C_{max}、AUC_{0-24}、T_{max}、$t_{1/2}$ 分别为：4.41±4.6、3.48±2.7（ng·mL^{-1}）；21.03±13.2、23.62±12.6（ng·h·mL^{-1}）；1.66±2.6、0.94±0.7（h）； 11.42±2.2、9.28±2.2（h）。末次 $AUC_{(0-24h)}$/首次 $AUC_{(0-24h)}$比值为 116.43±22.0%，说明 HIV 感染者体内阿兹夫定基本无蓄积。

2mg组和4mg组末次给药后Vd和Cl相比有显著统计学差异，4mg组T_{max}、C_{max}、AUC_{0-24h}高于2mg组，但相比无显著统计学差异。

用药后各组受试者 HIV 病毒载量符合对数正态分布，故对 HIV 病毒载量进行对数转换分析。2mg 组和 4mg 组用药后 HIV 病毒载量值均有显著降低，两组间差异无统计学意义。2mg 组和 4mg 组 HIV 病毒载量下降分别为 1.60～4.61 log_{10} copies/ml（2.56±0.96）、1.60～3.03 log_{10} copies/ml（2.49±0.45）。2mg 组和 4mg 组分别有 6 例（6/8）、7 例（7/8）受试者 HIV 病毒载量较基线下降＞0.5log_{10} copies/ml，分别有 6 例（6/8）、7 例（7/8）受试者 HIV 病毒载量较基线下降≥1log_{10} copies/ml。2mg 组有 1 例受试者 HIV 病毒载量较基线升高，治疗无效，考虑该受试者对 3TC 耐药，建议做耐药性检测，4mg 组全部受试者 HIV 载量较基线下降，治疗有效。

2mg 组和 4mg 组用药后 $CD4^+$ T 细胞计数有增加趋势，但差异无统计学意义。用药前后 $CD4^+$T 细胞计数差异两组间无统计学意义。2mg 组受试者给药后有 5 例（5/8）$CD4^+$T 细胞计数较基线上升，平均上升 155.31 个/ul，其中 4 例上升值＞50 个/ul。4mg

组受试者给药后有 5 例（5/8）$CD4^+T$ 细胞计数较基线上升，平均上升 221.88 个/ul，其中 4 例上升值＞50 个/ul。

1.3.3 药物药物相互作用临床研究总结

（1）FNC 与 TDF 相互作用临床研究

本研究共入组 15 例健康受试者，各组情况如下：

表 3　FNC 与 TDF 相互作用临床研究总体情况

分组	各组例数	给药剂量
FNC+TDF，FNC，TDF	5	
TDF，FNC+TDF，FNC	5	FNC4mg，TDF300mg
FNC，TDF，FNC+TDF	5	

FNC 组、TDF 组、FNC+TDF 组分别有 2 例、4 例、3 例受试者发生 4 次、4 次、3 次 AE，发生率为 13.3%、26.7%、20.0%，严重程度均为 1 级，均未给与治疗，未发生 SAE。FNC 组和 TDF 组 AE 均判定为与研究药物可能无关，除 TDF 组 1 例次失访外，其余均随访至消失/复常。FNC+TDF 组 AE 判定为与研究药物可能有关，表现为甘油三酯升高、乳酸脱氢酶升高、中性粒细胞绝对值降低，除 1 例次失访外，其余均随访至好转。

单独服用 FNC 的 $C_{max,ss}$、$AUC_{0-t,ss}$、$AUC_{0-\infty,ss}$、T_{max}、$T_{1/2}$ 分别为 2.43±1.75 $ng·mL^{-1}$、3.83±1.73 $ng·h·mL^{-1}$、5.61±0.86 $ng·h·mL^{-1}$、0.65±0.44 h、1.80±0.84 h，联合服用 FNC+TDF 后 FNC 的 $C_{max,ss}$、$AUC_{0-t,ss}$、$AUC_{0-\infty,ss}$、T_{max}、$T_{1/2}$ 分别为 1.50±0.80 $ng·mL^{-1}$、4.17±1.78 $ng·h·mL^{-1}$、5.60 $ng·h·mL^{-1}$、1.24±0.60 h、2.24 h。FNC+TDF/ FNC 的 $C_{max,ss}$、$AUC_{0-t,ss}$ 几何均数的比值分别为 90.11%和 286.28%，其 90%CI 分别为（55.50%, 146.32%）和（108.40%, 756.03%），提示多次联合服用 TDF 可显著增加 FNC 在体内的暴露程度。

FNC+TDF/TDF 的 $C_{max,ss}$、$AUC_{0-t,ss}$ 几何均数的比值分别为 82.45%和 94.57%，其 90%CI 分别为（69.91%, 97.24%）和（84.45%, 105.90%），提示多次联合服用 FNC 对 TDF 的药动学无显著影响。

FNC 与 TDF 的 C_{max} 和 $AUC_{0-t,ss}$ 的药代参数的相互影响可能与样本量较少有关系，不具有临床意义，故 FNC 合并使用 TDF 无需调整使用剂量。

（2）FNC 与 EFV 相互作用临床研究总结

本研究共入组 24 例健康受试者，各组情况如下：

表 4　FNC 与 EFV 相互作用临床研究总体情况

分组	各组例数	给药剂量
FNC/FNC+EFV	12	FNC4mg，EFV600mg
EFV/EFV+FNC	12	

FNC 组、EFV 组、FNC+EFV 组分别有 0 例、5 例、19 例受试者发生 0 次、5 次、33 次 AE，发生率为 0.0%、41.7%、79.2%，严重程度均为 1 级，均未给与治疗，未发生 SAE。FNC 组 AE 均判定为与研究药物肯定无关，均未给与治疗，均随访至消失/复常。FNC+EFV 组有 6 例受试者发生的 8 例次 AE 判定为与研究药物可能有关，其余均判定为与研究药物肯定无关/可能无关。FNC+EFV 组不良反应表现为血甘油三酯升高、丙氨酸氨基转移酶升高、低密度脂蛋白降低、高密度脂蛋白降低、心电图 PR 缩短、心电图异常、腹痛，除 2 例次失访外，其余均随访至好转或消失/复常。

单独服用 FNC 的 $C_{max,ss}$、$AUC_{0-t,ss}$、$AUC_{0-\infty,ss}$、T_{max}、$T_{1/2}$ 分别为：4.22±2.94 ng·mL^{-1}、6.78±4.83 ng·h·mL^{-1}、5.73±2.33 ng·h·mL^{-1}、1.14±0.74 h、1.02±0.36 h，联合服用 FNC+EFV 后 FNC 的 $C_{max,ss}$、$AUC_{0-t,ss}$、$AUC_{0-\infty,ss}$、T_{max}、$T_{1/2}$ 分别为 7.22±4.34 ng·mL^{-1}、14.04±7.96 ng·h·mL^{-1}、12.86±8.77ng·h·mL^{-1}、0.83±0.32 h、4.97±5.41 h。FNC+EFV/ FNC 的 $C_{max,\ ss}$、

$AUC_{0\text{-}t,ss}$、 $AUC_{0\text{-}\infty,\ ss}$ 几何均数的比值分别为 177.47%、231.5%、189.28%，其 90%CI 分别为（113.79%, 276.77%）、（142.98%, 375.00%）和（95.49%, 375.17%），提示多次服药时 EFV 可显著提高 FNC 在体内的暴露程度。EFV+FNC/EFV 的 $C_{max,\ ss}$、$AUC_{0\text{-}t,\ ss}$ 几何均数的比值分别为 84.37%和 90.00%，其 90%CI 分别为（43.69%, 162.91%）和（46.96%, 176.31%），但无法判断多次服药时 FNC 是否对 EFV 的药动学有显著影响。

FNC 与 EFV 的 C_{max} 和 $AUC_{0\text{-}t,ss}$ 的药代参数的相互影响可能与样本量较少有关系，不具有临床意义，故 FNC 合并使用 TDF 无需调整使用剂量。

1.3.4 阿兹夫定片餐后药代动力学临床研究总结

本研究共入组 12 例健康受试者，各组情况如下：

表 5　FNC 餐后药代动力学临床研究总体情况

分组	各组例数	给药剂量
空腹（餐后）	6	4mg
餐后（空腹）	6	

空腹给药组和餐后给药组分别有 4 例、3 例受试者发生 6 次、4 次 AE，发生率为 33.3%、25.0%，严重程度均为 1 级，均未给与治疗，未发生 SAE。所有 AE 均判定为与研究药物可能有关，均未给与治疗，因复查时间临近春节，受试者拒绝回院复查，未获受试者转归情况，空腹给药组 AE 表现为中性粒细胞绝对值下降、总胆红素升高、异常心电图、血乳酸升高、甘油三酯升高、胆固醇升高，餐后给药组 AE 表现为异常心电图、血乳酸升高和甘油三酯升高。

空腹给药的 C_{max}、$AUC_{0\text{-}t}$、$AUC_{0\text{-}\infty}$、$T_{1/2}$、T_{max} 分别为：3.10±2.20ng·mL^{-1}、3.59±1.77h·ng·mL^{-1}、5.20±1.36h·ng·mL^{-1}、1.28±0.78h、0.75±0.49h，餐后给药的 C_{max}、$AUC_{0\text{-}t}$、$AUC_{0\text{-}\infty}$、$T_{1/2}$、T_{max}、分别为：3.56±2.57ng·mL^{-1}、4.63±2.54h·ng·mL^{-1}、

6.52±2.24h·ng·mL^{-1}、1.35±0.61h、0.98±0.36h。餐后/空腹几何均数的比值分别为142.54%，163.71%和 114.92%，其中 90%CI 分别为（78.45%, 258.98%）、（109.92%, 243.80%）和（58.19%, 226.93%），表明餐后给药可提高 FNC 在体内的暴露程度，就餐对 FNC 的药动参数具有一定的影响。

1.3.5 阿兹夫定片在未接受过抗 HIV 病毒治疗的感染者中多次给药安全性的临床研究

本研究共入组 16 例未接受过治疗的 HIV 感染者，剂量组、各组例数及给药方法见表 6：

表 6 多次给药总体情况

试验组别	A	B
给药剂量	2mg	3mg
受试例数	8	8
给药方法	每天给药 1 次，每次 2 片，连续口服 7 天	每天给药 1 次，每次 3 片，连续口服 7 天

2mg 和 3mg 组分别有 1 例和 4 例受试者发生 1 次和 5 次 AE，严重程度为 1~2 级，均未给予治疗，未发生 SAE。2mg 组 AE 判定为与研究药物可能无关，均随访至消失。3mg 组有 3 例受试者发生的 3 例次 AE 判定为与研究药物可能有关，其余均判定为与研究药物可能无关。3mg 组不良反应表现为头晕、恶心，均随访至消失。

用药后各组受试者 HIV 病毒载量符合对数正态分布，故对 HIV 病毒载量进行对数转换分析。2mg 组和 3mg 组用药后 HIV 病毒载量值均有显著降低，两组间筛选期 HIV 病毒载量的差异无统计学意义，两组间停药 24h 后差异有统计学意义，两组间用药前后 HIV 病毒载量差值的差异有统计学意义。2mg 组 8 例受试者用药后 HIV 病毒载量较基线平均下降 1.67log_{10} copies/mL，3mg 组 8 例受试者用药后 HIV 病毒载量较基线平均下降 1.20log_{10} copies/mL。

2mg 组用药后 $CD4^+T$ 细胞计数有增加趋势，用药前后差异有统计学意义。3mg 组 $CD4^+T$ 细胞计数用药前后差异无统计学意义。用药前及用药后两组 $CD4^+T$ 细胞计数的差异均无统计学意义，但两组间用药前后 $CD4^+T$ 细胞计数差值的差异有统计学意义。2mg 组 8 例受试者用药后 $CD4^+T$ 细胞计数均较基线上升，平均上升 123.37cells/mm^3，7 例上升值>50cells/mm^3。3mg 组 6 例（6/8）受试者用药后 $CD4^+T$ 细胞计数较基线下降，平均下降 65.36cells/mm^3，2 例受试者用药后 $CD4^+T$ 细胞计数较基线上升（分别上升 45.66cells/mm^3 和 251.05cells/mm^3）。

1.4 II 期临床试验总结

本研究是一项多中心、随机、双盲双模拟、阳性对照、剂量探索 II 期临床研究，评价 FNC 联合逆转录酶抑制剂治疗未接受过抗 HIV 病毒治疗的感染者的安全性和有效性。试验在全国 5 个临床中心进行，筛选合格的受试者以 1:1:1:1 比例随机分配到 FNC 2mg 组、FNC 3mg 组、FNC 4mg 组、3TC 对照组，接受 48 周的抗病毒治疗和随访。共计入组 172 例受试者，其中 2 例受试者撤回知情同意书，15 例受试者因耐药、依从性不佳、自愿更换为自费药物、不良事件等原因退出研究，155 例完成 48 周访视。总体来说，受试者耐受性良好，对治疗方案的依从性高，发生的不良事件大部分为轻度或中度。

表 7 II 期临床试验给药方案

试验组别	研究药物	给药方案及周期
FNC 2mg 组	FNC 2 mg+ TDF+EFV	FNC 2 片+FNC 模拟片 2 片+3TC 模拟片 1 片+ TDF+EFV
FNC 3mg 组	FNC 3mg+ TDF+EFV	FNC 3 片+FNC 模拟片 1 片+3TC 模拟片 1 片+ TDF+EFV

FNC 4mg 组	FNC 4mg+TDF+EFV	FNC 4 片+3TC 模拟片 1 片+TDF+EFV
3TC 组	3TC+TDF+EFV	FNC 模拟片 4 片+3TC 1 片+TDF+EFV

每日一次口服 FNC 2mg/3mg/4mg+TDF+EFV 的治疗方案显示出确定的抗 HIV-1 疗效，能够显著抑制 HIV-1 的复制。FAS 集各组受试者接受治疗后，HIV-1 RNA 迅速降低，大部分受试者在 12~24 周 HIV-1 RNA 下降到 50copies/ml 水平以下直至 48 周。主要疗效指标 HIV-1 RNA＜50 copies/ml 的受试者百分率，FAS 集 FNC 2mg 组、FNC3mg 组、FNC4mg 组和 3TC 组分别为 88.1%、92.9%、93.0%和 90.7%，PPS 集分别为 97.2%、100%、100%和 97.5%，组间差异无统计学意义（P>0.05）。

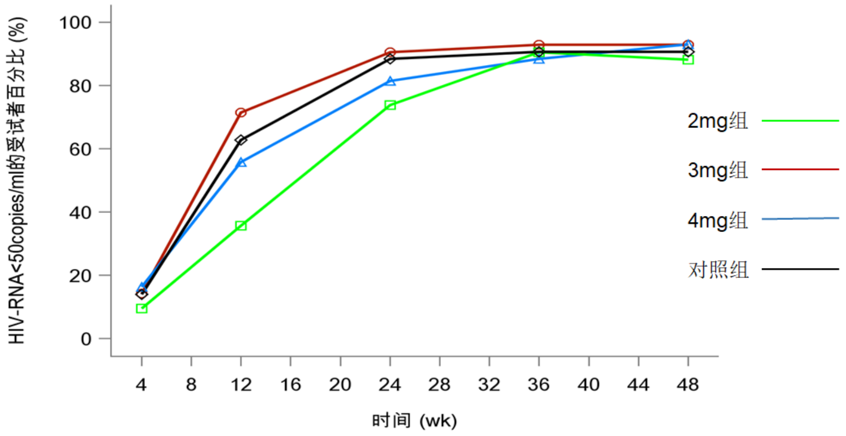

图 1 治疗后 HIV-1 RNA<50copies/ml 的受试者百分比历时性(FAS)

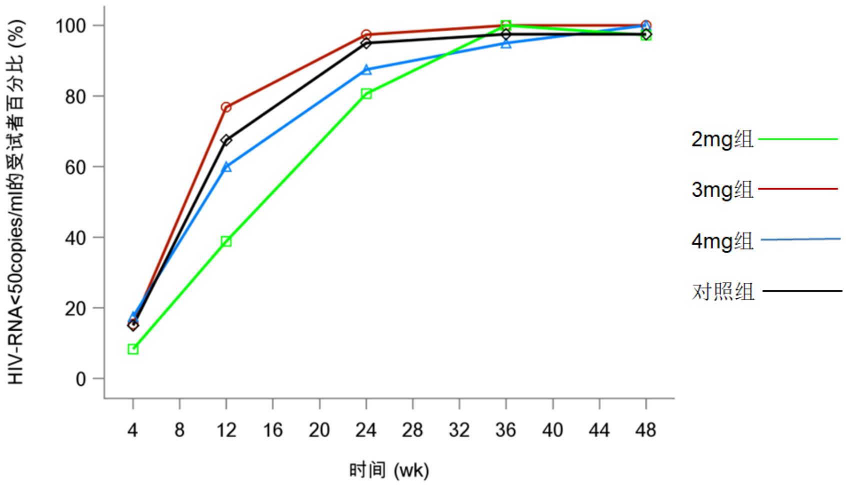

图 2 治疗后 HIV-1 RNA<50copies/ml 的受试者百分比历时性(PPS)

PPS 集 logistic 回归分析显示，受试者所在中心、组别、性别、基线 HIV-1 RNA 和 $CD4^+T$ 细胞计数水平对治疗 48 周后 HIV-1 RNA<50 copies/ml 百分率均无显著影响（P>0.05）。FNC+TDF+EFV 联合用药能有效控制 100,000 copies/ml 以上基线 HIV-1 RNA 感染者和 $CD4^+T$ 细胞计数低于 350 个/μl 感染者的病毒复制。

FAS 集和 PPS 集 HIV-1 RNA<50 copies/ml 受试者百分率的疗效历时性分析显示，治疗 12、24 周时，HIV-1 RNA<50 copies/ml 受试者百分率 FNC 3mg 略高于 FNC 2mg 组、FNC 4mg 组和 3TC 组，且 FNC 2mg 组与 FNC 3mg 组比较差异有统计学意义（P<0.05）。FNC 三个剂量组中 FNC 3mg 组的 HIV-1 RNA 下降速度较其他组相对较快。

三个次要疗效评价指标为治疗前后 HIV-1 RNA 对数值的变化、治疗前后 HIV-1 RNA 水平较基线下降≥1 log_{10} 的受试者数量及治疗前后 $CD4^+T$ 细胞计数的变化。各组受试者接受治疗后，HIV-1 RNA 迅速降低，大部分受试者在 4~12 周 HIV-1 RNA 下降 1.5~3 log_{10} copies/ml，并保持在此水平直至 48 周。治疗 48 周后，FAS 集 FNC 2mg 组、FNC 3mg 组、FNC 4mg 组和 3TC 组 HIV-1 RNA log_{10} copies/ml 变化值分别为 -3.313±0.6305、-3.149±0.5839、-3.082±0.7253 和-3.224±0.5087 log_{10} copies/ml；PPS 集分别为-3.476±0.4693、-3.215±0.4525、-3.188±0.5709 和-3.312±0.4051 log_{10} copies/ml，各组下降值均无明显差异。协方差分析扣除基线效应后，组间比较差异无统计学意义（P>0.05）。FAS 集疗效指标 HIV-1 RNA 下降≥1 log_{10} copies/ml 受试者百分率，FNC 2mg 组、FNC 3mg 组、FNC 4mg 组和 3TC 组分别为 100%、100%、97.7%和 100%； PPS 集各组均为 100%，组间差异无统计学意义（P>0.05）。FAS 集 FNC 2mg 组、FNC 3mg 组、FNC 4mg 组和 3TC 组 $CD4^+T$ 细胞计数变化值分别为 149.721±100.9320、117.676±113.7592、130.460±115.2048 和 142.163±110.2142 个/μL；PPS 集分别为 149.721±100.9320、117.676±113.7592、130.460±115.2048 和 142.163±110.2142 个/μL，

各组升高值相当。协方差分析扣除基线效应后，组间比较差异无统计学意义（P>0.05）。

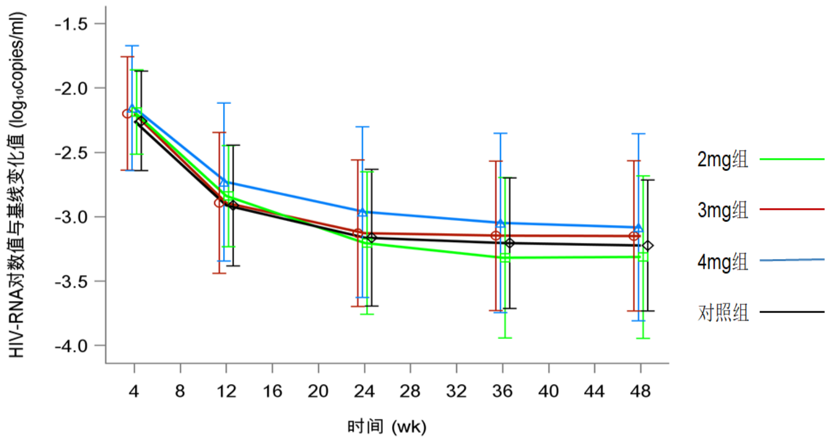

图 3 HIV-1 RNA 对数值(log_{10} copies/ml)与基线变化值(后-前)历时性(FAS) (Mean±SD)

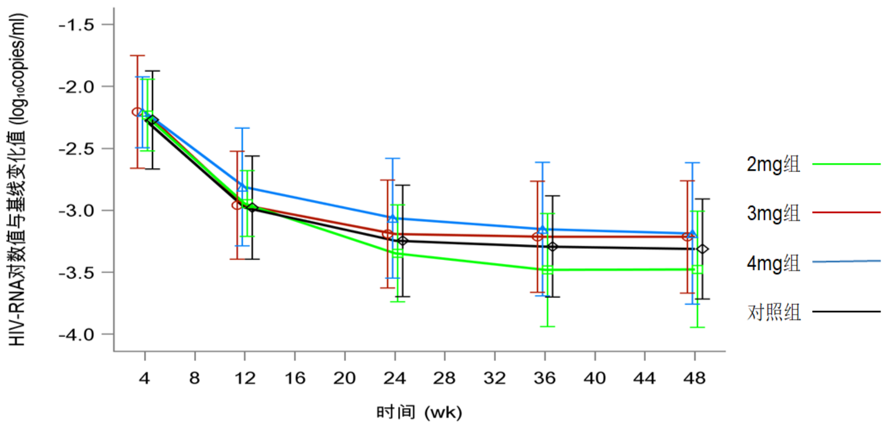

图 4 HIV-1 RNA 对数值(log_{10} copies/ml)与基线变化值(后-前)历时性(PPS) (Mean±SD)

FNC 2mg 组、FNC 3mg 组、FNC 4mg 组和 3TC 组平均暴露量相近，FNC 的平均药物暴露量分别为 1187.8±376.74、1273.6±248.37、1267.9±271.02、1249.1±325.86 片，表明有足够的药物暴露对 FNC 的安全性进行评价。

FNC 2mg 组、FNC 3mg 组、FNC 4mg 组显示了良好的安全性，与药物有关的不良事件（不良反应）总发生率（85.7%、92.9%、93.0%）和严重程度与 3TC 组（88.4%）相似。试验期间未发生死亡事件，仅 3TC 组 1 例受试者发生与药物可能无关的严重不良事件（12.3%）。在所有发生的不良反应中，导致受试者脱落的不良反应仅 3 例，分别为 FNC 3mg 组（2.4%）、FNC 4mg 组（2.3%）、3TC 组（4.7%）各 1 例，组间比

较差异无统计学意义（P>0.05）。

发生率最高的不良反应为头晕（38.1%、59.5%、51.2%、53.5%），其次为丙氨酸氨基转移酶升高（35.7%、23.8%、41.9%、25.6%）、γ-谷氨酰转移酶升高（14.3%、11.9%、32.6%、9.3%）、天门冬氨酸氨基转移酶升高（16.7%、11.9%、20.9%、14.0%）、血尿酸升高（14.3%、11.9%、16.3%、14.0%）。FNC 三个剂量组中 FNC 3mg 组头晕的不良反应发生率略高于 3TC 组，其他常见不良反应发生率略低于 3TC 组或与之相近。各组间不良反应发生率相近，没有明显差异。

头晕是发生率最高的不良反应，FNC 各剂量组间与 3TC 组的不良反应发生率相近。头晕不良反应主要表现为 1~2 级的轻度升高，3 级仅在 FNC 3mg 组和 3TC 组发生，发生率分别为 2.4%、4.7%，未发生 4 级头晕不良反应。参考 EFV 说明书中关于不良反应的描述，发生率高于 5%且与治疗有关的中重度常见不良事件头晕为 8.5%，故考虑研究期间发生的头晕不良反应一定程度上与每日服用的背景治疗药物 EFV 有关。

抗病毒治疗过程中，肝损伤也是常见的不良反应。肝功能指标异常（如 AST、ALT、γ-GT 升高），也是发生率较高的不良反应。AST、ALT、γ-GT 升高不良反应主要表现为 1~2 级，3 级仅在 FNC 3mg 组发生（4.8%，2/42），未发生 4 级肝功能指标异常的不良反应。参考 TDF 说明书中关于不良反应的描述，未接受过抗逆转录酶病毒治疗的受试者接受 TDF+EFV+3TC 联合用药，观察到的 3 级/4 级实验室异常不良反应中，AST 发生率 5%、ALT 发生率 4%；参考 EFV 说明书中关于不良反应的描述，接受 600mg EFV 的患者中有 3%AST 和 ALT 升高到正常值上限的 5 倍以上，有 4%患者 γ-GT 升高到正常值上限的 5 倍以上。总体上比较，FNC 三个剂量组中 FNC 3mg 组肝功能异常的不良反应发生率略低于 3TC 组。

2 级不良反应发生率最高的为血磷降低，FNC 2mg 组、FNC 3mg 在、FNC 4mg 组、3TC 组发生率分别为 9.5%、4.8%、11.6%、7.0%。据文献报道，长期服用 TDF 有潜在的肾毒性，包括急性肾功能衰竭和范科尼综合征（伴有严重低磷血症的肾小管损伤），其最常见和最早期的表现是血磷降低，血磷降低的不良反应发生率大约为 4%，故考虑本研究期间发生的血磷降低不良反应一定程度上与每日服用的背景治疗药物 TDF 有关。发生的血尿酸升高不良反应也是已被报道的抗病毒治疗中常见的不良反应。未发生中重程度及严重的非预期不良反应。

治疗后血常规、尿常规、血生化、心肌酶谱、血乳酸、生命体征、体格检查、心电图、腹部 B 超、胸部 X 线检查，仅个别受试者的个别指标发生异常有临床意义的变化，各项检查四个治疗组出现异常变化的指标及发生率相近，无明显差异。未发生肾小球滤过率异常的不良反应。治疗期间，四个治疗组肾小球滤过率的均值均在正常范围内，未见有临床意义的组间差别。

FNC 联合 TDF 和 EFV 组合治疗未接受过抗 HIV-1 感染者 48 周，可以显著抑制 HIV-1 的复制，HIV-1 RNA 显著下降，治疗效果与目前推荐的一线治疗方案 3TC+TDF+EFV 相当，且 FNC 3mg 组体现了较快的 HIV-1 RNA 下降速度；安全性良好，FNC 三个剂量组与 3TC 组在不良反应总发生率和严重程度上相似，FNC 3mg 组较 3TC 组体现了略低的肝功能异常的不良反应发生率。

2 研究目的

在临床常规治疗的基础上，评价阿兹夫定片治疗新型冠状病毒感染肺炎的有效性和安全性。

3 研究设计

3.1 总体设计

本研究采用随机、开放、对照临床试验设计。本研究计划纳入 20 例符合入组条件的新型冠状病毒感染的肺炎患者，按 1:1 比例随机分配到试验组或对照组，每组 10 例。试验组接受常规治疗和阿兹夫定片，对照组接受常规治疗。

本研究共分为三个阶段：筛选期、治疗期、随访期，见下图：

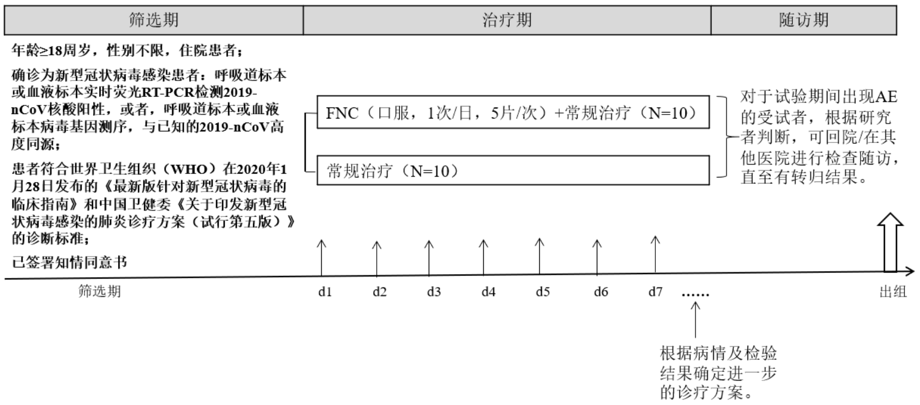

图 5 研究流程示意图

3.2 随机方法和随机表

- ✓ 本研究由随机表确定受试者接受试验组或对照组。
- ✓ 随机表应用 SAS 按 1:1 随机产生。每例筛选期检查合格的受试者获得随机号，根据预先制定的随机表随机分配进入试验组或对照组，并按照相应的治疗方案接受治疗。

3.3 终止标准

如理由充分，本研究可提前终止或暂停，其原因包括但不限于：

（1）试验中发生严重安全性问题，由研究者讨论后决议终止/暂停试验。

（2）在研究中发现临床研究方案有重大失误，难以评价药物效应；或者一项设

计较好的方案，在实施中发生了重要偏差，再继续下去难以评价药物效应。由研究者讨论后决议终止/暂停试验。

（3）伦理委员会要求终止已经批准的研究。

4 受试者选择

研究者必须保证所有参与研究的受试者符合以下入选标准，同时不符合任何一条排除标准。

4.1 入选标准

（1） 年龄≥18 周岁（含临界值），性别不限；

（2） 呼吸道标本或血液标本实时荧光 RT-PCR 检测 2019-nCoV 核酸阳性，或者，呼吸道标本或血液标本病毒基因测序，与已知的 2019-nCoV 高度同源；

（3） 确诊为新型冠状病毒感染者，患者符合世界卫生组织（WHO）在 2020 年 1 月 28 日发布的《最新版针对新型冠状病毒的临床指南》和中国卫健委《关于印发新型冠状病毒感染的肺炎诊疗方案（试行第五版）》的诊断标准；

（4） 已签署知情同意书。

4.2 排除标准

（1） 已知或怀疑对阿兹夫定片的组成成分过敏；

（2） 合并有吸收障碍综合征，或其它任何对胃肠道吸收产生影响的状况，需要静脉营养或不能口服药物的患者；

（3） 正在接受抗 HIV 病毒治疗的患者；

（4） 患者出现以下情况之一者：出现呼吸衰竭，且需要机械通气；出现休克；合并其他器官功能衰竭需 ICU 监护治疗；

（5） 妊娠期、哺乳期妇女或在试验期间及结束 6 个月内有生育计划；

（6） 给药前 12 周内参加过其它临床试验或者正在使用试验性药物；

（7） 根据研究者的判断，具有不适合参加本试验的其它情况。

4.3 退出标准

不论是否服用了研究药物，对因任何原因退出试验的受试者都将保留其编号。

4.3.1 研究者决定的退出

是指已经入选的受试者在试验过程中出现不宜继续进行试验的情况下，研究者决定该受试者退出本研究。

（1） 受试者依从性差，不能按照试验方案执行。

（2） 受试者在试验期间发生妊娠，经研究者确认后，分析获益风险情况，可退出试验。

（3） 受试者发生药物不良事件，经分析处理不适宜继续参加试验。

（4） 研究者认为受试者出现不适宜继续参加试验的其它情况。

4.3.2 受试者自行退出试验

（1） 受试者有权因任何原因中途退出试验；

（2） 受试者虽未明确提出退出试验，但不再继续按照试验方案的规定接受访视或接受研究药物或配合检测，并且研究者在本次访视期至下次访视期间无法联系上受试者时，受试者以失访计，该种失访也属于“退出”（或称“脱落”）。

5 受试者的治疗

5.1 研究药物

药物名称、规格、包装

通用名：阿兹夫定片（Azvudine，FNC）

剂型：片剂

规格：1mg/片

保存条件：密封、室温保存

有效期：2 年

5.2 治疗方案

筛选合格的受试者被分配到试验组或对照组，组别见表 9：

表 9 各试验组设计例数与给药周期

组别	治疗方案	受试者例数	给药周期
试验组	阿兹夫定片+常规治疗	10	每日给药直至受试者解除隔离出院或根据病情转至相应科室治疗其他疾病或病死
对照组	常规治疗	10	

阿兹夫定片：受试者口服给药，每日服药一次，每次5片。

常规治疗：参照中国卫健委《关于印发新型冠状病毒感染的肺炎诊疗方案（试行第五版）》，给予受试者相应的治疗措施。

5.3 合并用药

在整个试验期间，研究者将记录关于伴随疾病和包括药物治疗在内的任何治疗性干预、外科手术等方面的信息。在可能的情况下还应包括所有疾病的诊断和发病日期及症状缓解的日期、以及包括药物的名称、服药日期和手术的描述等。

（1）允许使用的治疗

抗病毒治疗过程中，肝损害是常见的不良反应。试验期间，如果发生肝损害，

需要及时给与保肝治疗。

（2）禁用使用的治疗

试验期间，受试者不得使用其它抗 HIV 病毒药物，以及研究者禁止使用的其它药物。

6 试验步骤

6.1 有效性及安全性观察指标

表 10 受试者观察指标具体要求

项目	要求及观察指标
人口学资料	民族、年龄、性别、身高、体重
问诊	既往/现病史（疾病史、手术史）、过敏史、用药史、酗酒史、药物滥用史、临床试验参加史、手术治疗史、妊娠/哺乳情况
生命体征	体温、心率、呼吸、血压
指脉氧	——
动脉血气分析	氧分压、氧饱和度
病原核酸检测	呼吸道标本/血液标本
心电图	12 或 18 导联心电图
肺部 DR/CT	——
白介素 6	——
血常规	红细胞计数（RBC）、血红蛋白（Hb）、红细胞比积（HCT）、平均红细胞体积（MCV）、平均红细胞血红蛋白含量（MCH）、平均红细胞血红蛋白浓度（MCHC）、白细胞计数（WBC）、嗜中性粒细胞绝对数（NEUT#）、淋巴细胞绝对数（LYMPH#）、单核细胞绝对数（MONO#）、嗜酸性粒细胞绝对数（EOS#）、嗜碱性粒细胞绝对数（BASO#）、血小板计数（PLT）

血生化	天门冬氨酸氨基转移酶（AST）、碱性磷酸酶（ALP）、胆碱酯酶（CHE）、丙氨酸氨基转移酶（ALT）、白蛋白（Alb）、总蛋白（TP）、总胆红素（TBIL）、直接胆红素（DBIL）、谷氨酰基转移酶（GGT）、肌酐（Scr）、尿素（UREA）、尿酸（UA）
血沉	血沉（ESR）
C-反应蛋白	C-反应蛋白（CRP）
心肌标志物	肌钙蛋白 I、B 型钠尿肽前体（BNP）
降钙素原	降钙素原（PCT）
血乳酸	——
心肌酶谱	肌酸激酶（CK）、肌酸激酶同工酶（CK-MB）
凝血功能	血浆凝血酶原时间测定（PT）、活化部分凝血活酶时间测定（APTT）、血浆纤维蛋白原（FIB）测定、凝血酶时间测定（TT）
传染五项	甲型肝炎抗体、乙肝表面抗原（HBsAg）、丙型肝炎病毒抗体（HCV 抗体）、人体免疫缺陷病毒抗原抗体（HIV 抗体）、梅毒螺旋体抗体（Anti-TP）
免疫五项	IgA、IgG、IgM、补体 C3 和 C4
呼吸道病毒九项	嗜肺军团菌、肺炎支原体、Q 热立克次体、肺炎衣原体、腺病毒、呼吸道合胞病毒、甲型流感病毒、乙型流感病毒、副流感病毒
快速呼吸道病毒三联检	合胞病毒、腺病毒、肺炎支原体
T 淋巴细胞亚群	$CD4^{+}$T 细胞计数
血妊娠	适用于女性受试者，可接受不超过 72h 的检查结果

6.2 有效性及安全性观察时点

6.2.1 流程图

表 11 临床研究流程图

研究阶段	筛选期	治疗期[3]								出组检查/提前退出
时间段/点		d1	d2	d3	d4	d5	d6	d7	……[4]	
签署知情同意书	X									
核查入选/排除标准	X									
人口学资料	X									
问诊	X									
主诉症状		X	X	X	X	X	X	X		X
生命体征	X	X	X	X	X	X	X	X		X[1]
指脉氧		X	X	X	X	X	X	X		
血常规	X			X		X		X		X[1]
血沉	X			X		X		X		X[1]
白介素 6	X			X		X		X		X[1]
C-反应蛋白	X			X		X		X		X[1]
血生化	X			X		X		X		X[1]
凝血功能	X			X		X		X		X[1]
心肌标志物	X			X		X		X		X[1]
降钙素原	X			X		X		X		X[1]
心肌酶谱	X			X		X		X		X[1]
动脉血气分析	X			X		X		X		X[1]

血乳酸	X							X		X[1]
传染五项	X									
免疫五项	X									
呼吸道病毒九项	X									
快速呼吸道病毒三联检	X									
T 淋巴细胞亚群	X					X				X
血妊娠 [2]	X									X[1]
心电图	X							X		X[1]
肺部 DR/CT	X			X		X		X		X[1]
病原核酸检测	X			X		X		X		X
随机分组		X								
给药		X								
合并用药		X	X	X	X	X	X	X	X	X
不良事件		X	X	X	X	X	X	X	X	X[1]

1：如有异常，应进行随访。不良事件应随访至恢复或到稳定状态等明确结局，详细结局状态请参照本方案“6.3 不良事件的随访”。

2：仅适用于育龄女性患者。

3：根据受试者病情及检验结果研究者可调整下一步诊疗方案（有些病人进展很快，需严密观察），治疗过程中如出现危重型或疾病进展快的指标，由研究者判断是否申请专家会诊。

4：根据受试者病情及检验结果确定进一步的诊疗方案。

6.2.2 研究步骤

（1）首次给药当天记为第 1 天（d1）。

（2）在受试者签署书面知情同意书之前，已有本研究中心出具的筛选前 14 天内以下检查项结果，可接受作为筛选期检查结果：血常规、血沉、白介素 6、C-反应蛋白、血生化、凝血功能、心肌标志物、降钙素原、心肌酶谱、动脉血气分析、血乳酸、传染五项、免疫五项、呼吸道病毒九项、快速呼吸道病毒三联检、T 淋巴细胞亚群、心电图、肺部 DR/CT、病原核酸检测。

6.3 不良事件的随访

对于试验期间出现 AE 的受试者，根据研究者判断，可回院/在其他医院进行检查随访，直至以下任一种情况发生：

- 完全恢复或恢复至基线水平；
- 研究者确认该 AE 已稳定、预期不会进一步改善；
- 受试者死亡或失去联络。

7 终点指标

7.1 疗效指标

（1）体温恢复正常的时间和比例；

（2）呼吸道症状及体征（肺部啰音、咳嗽、咯痰、咽痛等）改善的时间和比例；

（3）腹泻、肌痛、乏力等其他症状改善的时间和比例；

（4）肺部影像学改善的时间和比例；

（5）2019-nCoV 核酸转阴的时间和比例；

（6）氧合指数改善的时间和比例；

（7）CD4 计数改善的时间和比例；

（8）轻型/普通型转重型比例，重型转危重型比例；

（9）住院时间；

（10） 死亡率。

7.2 安全性指标

（1） 生命体征（体温、心率、呼吸、血压）；

（2） 血常规、血沉、白介素 6、C-反应蛋白、肝功能、肾功能、凝血功能、血乳酸、心肌酶谱、心肌标志物、降钙素原、妊娠试验；

（3） 心电图；

（4） 不良事件：包括类型、发生率、严重程度、时间和药物相关性，严重程度参考 CTCAE（5.0 版）进行评估。

8 安全性评价

8.1 安全性评价参数

与研究药物有关的不良事件；严重不良事件；实验室检查值异常；非预期安全性事件。特别关注的不良事件的临床表现特征、严重程度、发生时间、持续时间、处理方法及预后。

8.2 安全性评估

8.2.1 临床安全性评估

通过受试者自发报告或医师直接观察或通过非诱导的方式询问受试者有关不良事件情况评价其临床安全性。

8.2.2 实验室安全性评估

研究者将在治疗前后对受试者进行血常规、血沉、白介素6、C-反应蛋白、血生化、凝血功能、心肌标志物、降钙素原、心肌酶谱、动脉血气分析、血乳酸、T淋巴细胞亚群、血妊娠、心电图、肺部DR/CT、病原核酸检测等检查，以评价其安全性。

8.3 不良事件

8.3.1 定义

不良事件（AE），此处指临床试验受试者接受试验药物后出现的所有不良医学事件，但不一定能推论出与试验用药品有明确的因果关系。不良事件可以是症状体征、疾病或实验室检查异常，包括以下几种情况：

（1）原有的（进入临床试验之前）医学状况/疾病的加重（包括症状，体征，实验室异常的加重）；

（2）新发生的任何不良事件：新发生的任何的不良医学状况（包括症状，体征，新诊断的疾病）；

（3）异常的具有临床意义的实验室检查值或者结果，且不是由伴随疾病导致。

不良反应（ADR）：指临床试验中发生的任何与试验用药物可能有关的对人体有害或者非预期的反应。试验用药物与不良反应之间的因果关系至少有一个合理的可能性，即不能排除相关性。

重要不良事件：是指除SAE外，发生的任何导致必须采用针对性医疗措施（如停药、降低剂量和对症治疗）的AE和血液学或其他实验室检查明显异常。

严重不良事件（SAE）：指因使用任何剂量的试验用药品发生的、任何引起人体损害的不利医学事件：

- ✓ 导致死亡；
- ✓ 危及生命（定义中的"危及生命"是当其发生时会造成受试者立即死亡的AE，而不包括进一步发展后才会引起死亡的AE）；
- ✓ 受试者需要住院治疗或延长住院时间；
- ✓ 导致永久的或严重的残疾/功能丧失；
- ✓ 导致先天性异常/出生缺陷。
- ✓ 其他重要医学事件：必须运用医学和科学的判断决定是否对其他的情况加速报告，如重要医学事件可能不会立即危及生命、死亡或住院，但如需要采取医学措施来预防如上情形之一的发生，也通常被视为是严重的。这些事件的例子包括但不限于下列事件：需要在急诊室或家中强化治疗的过敏性支气管痉挛、不需要住院治疗的血液系统恶病质或惊厥、潜在的药物性肝损伤、病原体（如致病性或非致病性病原体）通过研究药物的可疑传播、妊娠、药物过量、继发肿瘤等。

以下情况不作为SAE报告：

（1）与不良事件恶化无关的住院治疗或住院时间延长本身不是SAE。例如：

- 因原有疾病入院，并没有新的AE的发生，也没有原有疾病的加重（如：为了检查试验前至今持续存在的实验室检查异常）；
- 管理原因的住院（如：每年例行的体检）；
- 临床试验期间试验方案规定的住院（如：按试验方案的要求进行操作）；
- 与AE恶化无关的择期住院（如：择期整容手术）；
- 已预定的治疗或外科手术应在整个试验方案和/或受试者个人的基线资料中予以记录；

- 仅因为血液品使用而入院。

（2）受试者因行政性和社会目的而入院的情况不作为 AE 报告，例如受试者住院疗养，或由于医保报销的原因而住院。

（3）诊断性或治疗性的侵入性（如手术）、非侵入性操作不应作为 AE 报告。但导致此项操作的疾病状况符合 AE 的定义时，应予以报告，如 AE 报告期间发病的急性阑尾炎应报告为AE，而因此进行的阑尾切除术应记录为该AE的治疗方法。

（4）临床研究中导致住院治疗或住院时间延长的不良事件应视为 SAE。住院不包括以下情况：康复机构；疗养院；常规急诊室收治；当日手术（如门诊/当日/非卧床的手术）。

（5）如果试验期间因疾病进展的症状和体征而住院不应作为 SAE 报告，但因疾病进展（包括进展的症状和体征）而死亡应作为 SAE 报告。试验期间如果癌症的最终结果为死亡，那么导致死亡的事件必须作为严重不良事件报告。

8.3.2 不良事件的收集与记录

在整个研究过程中对不良事件进行监测，研究者有责任记录在研究期间观察到的所有AE。本研究应记录从首次给药开始直至随访结束，期间发生的任何不良医学事件，不论严重程度以及与研究药物的因果关系，研究者均需要在病历中相应的AE页中进行记录。

8.3.3 不良事件严重程度分级标准

本研究 AE 严重程度是根据常见不良反应时间评价标准（CTCAE v5.0）分级，AE 强度的分级如下：

1 级：轻度；无症状或轻微；仅临床或诊断所见；无需治疗。

2 级：中度；需要较小、局部或非侵入性治疗；与年龄相当的工具性日常生

活活动受限（工具性日常生活活动是指做饭、购买衣物、使用电话、理财等）。

3 级：严重或具重要医学意义，但不会立即危机生命；导致住院或延长住院时间；致残；自理性日常生活活动受限（自理性日常生活是指洗澡、穿脱衣、吃饭、盥洗、服药，并未卧床不起）。

4 级：危及生命，需紧急治疗。

5 级：与 AE 相关的死亡。

8.3.4 不良事件与研究药物相关性评估

（1）不良事件与研究药物关联性的分析需综合考虑以下因素：

①不良事件是否与研究药物起作用的时间有合理的时间顺序；

②研究药物停用或减量后，该不良事件是否消失或减轻；

③再次用药，该不良事件是否再现；

④不良事件的临床或病理学表现是否与已知的研究药物或药物的分类药理学及毒理学的知识相符；

⑤不良事件是否可用原先的疾病或患者本人或环境因素等解释。

（2）不良事件与研究药物关联性有以下几种情况：

①肯定有关：具有使用研究药物的证据，不良事件的发生与研究药物使用有合理的时间顺序，不良事件以研究药物解释比其它原因解释更为合理。停药反应阳性，重复用药（如果可行）反应阳性。

②很可能有关：具有使用研究药物的证据，不良事件的发生与研究药物的使用有合理的时间顺序。不良事件以研究药物解释比其它原因解释更为合理。停药反应阳性。

③可能有关：具有使用研究药物的证据，不良事件的发生与使用研究药物

在时间上的相关性合理。不良事件可用其它原因解释。停药反应阳性。

④可能无关：具有使用研究药物的证据，发生的不良事件也许用其它原因更能解释。停药反应阴性或不明确。

⑤肯定无关：未使用研究药物，或使用研究药物和不良事件发生的时间无相关性，或另有明确导致不良事件的原因。

表 12 不良事件与研究药物关系的判定

	肯定有关	很可能有关	可能有关	可能无关	肯定无关
与研究药物有合理的时间顺序	＋	＋	＋	＋	－
已知的药物反应类型	＋	＋	＋	－	－
停药或减量后反应减轻或消失	＋	＋	±	±	－
再次给药后反应反复出现	＋	?	?	?	－
无法与用药作用、患者病情的进展、其他治疗的影响来解释	＋	＋	－	±	－

注： ＋肯定 －否定 ±有可能 ？未知 肯定、很可能、可能计为药物不良反应，统计不良反应发生率。

8.3.5 严重不良事件报告

本研究应记录从首次给药开始直至随访结束。如果受试者在试验过程中发生SAE，无论是否与研究药物有关，研究者应立即对受试者采取适当的治疗措施，保障受试者的安全，及时报告所在中心的临床试验负责人。研究者获知 SAE 后24h 内报告给申办者、监管部门、伦理委员会。对于信息暂时不完整不确定的 SAE，也应按照 GCP 的原则及时报告，待后续获得更多信息后再以随访报告的形式补充报告。

研究者要向申办者提交随访报告，直到不良事件严重不良事件痊愈、好转、

稳定、得到合理的解释、受试者死亡、失访。如果是永久性损害，要一直随访到认为不良事件已经稳定。由于疾病进展导致的死亡可以豁免死亡报告。

8.4 妊娠

试验期间，如果服用研究药物的女性受试者或男性受试者伴侣怀孕，妊娠将会被记录并报告给伦理委员会和申办者。任何在参与本研究期间怀孕（宫内）的女性受试者必须退出本研究并立即停止服用研究用药，发生的任何妊娠事件使用临床试验妊娠表进行报告。对于发生妊娠事件的女性受试者或男性受试者的伴侣必须随访直至婴儿分娩，以确定妊娠结果（包括提前终止妊娠）和母婴状况。妊娠并发症和出于医学原因的选择性终止妊娠必须作为 AE 或 SAE 报告，自然流产必须作为 SAE 报告。

9 数据管理和统计分析

9.1 数据管理

9.1.1 病例报告表记录要求

（1）CRF：全部病例，包括脱落病例，按照原始记录，认真填写病例报告表。数据测量的研究者对数据的真实性负责。

（2）原始化验单：必须齐全。病例报告表所记录的实验室检查数据或描述，结果要与原始检验报告一致。监查员应对此一致性负责。

（3）实验室检查异常有临床意义的数据：无论给药前后，研究者均应加以核实，及时复查，确属异常者。

（4）CRF 填表说明：见病例报告表。

9.1.2 数据管理采集和审核

申办者将对指定的研究人员进行 CRF 填写培训。相关人员接受过培训后，按照试验方案要求，将数据填入本项目指定的 CRF 中，最后需要主要研究者或其授权的研究人员进行签名。

临床监查员应及时对研究人员填写的 CRF 数据进行完整性和真实性审核，必要时提出质疑请研究者进行核实修改，直至数据质疑消除。

医学审核人员应及时对研究人员填写的 CRF 数据进行医学专业审核，必要时提出质疑请研究者进行核实修改，直至数据质疑消除。

9.2 统计分析

（1）统计软件

- 临床部分统计分析采用 SAS 9.4（或更高版本）完成。

（2）统计描述

- 计量数据采用均数、标准差、中位数、最大值、最小值描述。
- 计数数据采用频数、百分比描述。

（3）分析人群

本研究的分析人群包括全分析集（FAS）、符合方案集（PPS）。

- 全分析集（FAS）：所有经随机化入组，至少使用一次研究药物的病例集合。主要疗效指标缺失时，根据意向性分析（intention to treat，ITT 分析），用前一次结果结转。FAS 为主要分析集。次要疗效指标根据 FAS 中实际数据分析。
- 符合方案集（PPS）：是由充分依从于试验方案的受试者所产生的数据集，依从性包括所接受的治疗、主要终点指标测量的可获得性以及对试验方案没有大的违背等。PPS 分析用于主要疗效指标。

（4）基线分析

- 在 FAS 和 PPS 中进行，分析内容包括病例分布情况、人口学资料、基线分析。

（5）疗效分析

在 FAS 和 PPS 中进行，对两组的体温恢复正常的时间和比例；呼吸道症状及体征（肺部啰音、咳嗽、咯痰、咽痛等）改善的时间和比例；腹泻、肌痛、乏力等其他症状改善的时间和比例；肺部影像学改善的时间和比例；2019-nCoV 核酸转阴时间和比例；氧合指数改善的时间和比例；CD4 计数改善的时间和比例；轻型/普通型转重型比例，重型转危重型比例；住院时间；死亡率进行组间比较。

（6）安全性分析

- 用安全性数据集（SS）。
- 不良事件将使用 MedDRA（国际医学用语词典）进行编码。不良事件的分析将基于治疗中出现的不良事件（TEAE）。TEAE 定义为治疗后发生或恶化的不良事件。对各治疗组中的 TEAE，按系统器官分类（SOC）和首选术语（PT）总结分析，计算发生人数和发生率。
- 使用描述性统计量对生命体征和实验室检查进行总结。使用移位表描述在用药前后实验室检查正常/异常结果的变化。

10 研究管理

10.1 伦理委员会

在试验开始前，研究者/研究单位应取得伦理委员会对试验方案、知情同意书及其他可能需要提交的文件的批准意见。

在试验进行期间，如果试验方案、知情同意书等有任何新增修订，都应进行伦理审批或备案。

10.2 知情同意

研究者必须向受试者说明参加临床试验是自愿的，而且在试验的任何阶段有权随时退出试验而不会遭到歧视和报复，其医疗待遇和权益不受影响。必须使受试者了解到参加试验及在试验中的个人资料均属保密。还需告知受试者临床试验的背景、试验目的、试验用药物的信息、试验流程、预期可能的受益和可能发生的风险和不便，使受试者有充分的时间考虑是否愿意参加试验。

受试者或其法定代理人、执行知情同意过程的研究者或其代表均需在知情同意书上签名并签署日期。

10.3 资料的保存

试验资料应保留至试验结束后 5 年，但若现行法规或与申办者的协议中有要求，这些资料还应保存更长的时间。申办者将以书面形式通知研究者这些资料何时将不再需要保存。

10.4 研究认可

研究单位的主要研究者和申办者应在本方案签名页上签名，证明他/她已同意本研究方案的内容，并将依照此方案开展、实施本项研究。

11 参考文献

[1] 世界医学协会赫尔辛基宣言[Z], 2013-10.

[2] 国家药品监督管理局. 药品注册管理办法 局令第 28 号[Z] , 2007-7-10.

[3] 国家药品监督管理局. 药物临床试验质量管理规范[Z], 2003-6-4.

[4] 新型冠状病毒感染的肺炎诊疗方案（试行第五版）.

[5] WHO, Clinical management of severe acute respiratory infection when novel coronavirus (nCoV) infection is suspected.

[6] Nanshan Chen, Min Zhou. Epidemiological and clinical characteristics of 99 cases of 2019 novel coronavirus pneumonia in Wuhan, China: a descriptive study, Articles, 2020.

[7] Roujian Lu, Xiang Zhao. Genomic characterisation and epidemiology of 2019 novel coronavirus: implications for virus origins and receptor binding, Articles, 2020.

阿兹夫定片治疗新型冠状病毒感染肺炎的随机、开放、对照临床试验

A randomized, open-label, controlled clinical trial of Azvudine tablets in the treatment of Coronavirus disease 2019

知情同意书

The written informed consent for the enrolled participants

研究申请者： 光山县人民医院

研究负责单位： 光山县人民医院

研究中心地址： 河南省光山县正大街 10 号

主要研究者： 裴广忠

伦理委员会联系人： 任志刚

联系电话及邮箱： 18703636245；fccrenzg@zzu.edu.cn

知情同意书•知情告知页

受试者须知

尊敬的女士/先生：

我们邀请您参加一项阿兹夫定片治疗新型冠状病毒感染肺炎的随机、开放、对照临床试验。在您决定是否参加这项试验之前，请尽可能仔细阅读以下内容，它可以帮助您了解该项试验以及为何要进行这项试验、试验的程序和期限、参加试验后可能给您带来的益处、风险和不适。如果您愿意，您也可以和您的亲属、朋友一起讨论，帮助您做出决定。如果有不清楚的地方，您可以向您的负责医生咨询。一旦您所有的问题都被解答、您对有关这项研究的解释说明很满意、且您决定参加，您将被要求签署这份知情同意书。参加研究是您的自愿行为，您可以同意参加，也可以不同意。

一、 研究背景

2019 新型冠状病毒（2019-novel coronavirus，2019-nCoV）属于新发现的β属冠状病毒。2019-nCoV 感染肺炎疫情已是国际关注的突发公共卫生事件。新型冠状病毒感染肺炎可引起人呼吸道症状、发热、咳嗽、气促和呼吸困难等，在危重病例中，病毒主要导致严重的全身炎症反应（细胞因子风暴），引起急性肺损伤、重症肺炎、急性呼吸窘迫综合征的（ARDS）、严重低氧血症、感染性休克，肾衰竭，甚至死亡，目前该病毒已表现出强的传染性。

冠状病毒的复制生命周期中的不可缺少的一个环节是病毒的 RNA 的合成。通常病毒 RNA 的复制利用天然核苷为底物完成。但是，快速复制的病毒 RNA 对底物的选择性显著降低，极其容易错误地将人工合成的核苷结合进入病毒 RNA，使其病毒 RNA 链的合成终止，达到杀死病毒的结果。

核苷在抗病毒感染的治疗中起着至关重要的作用。如抗艾滋病药中有 7 款核苷药，如 3TC，FTC,TDF 或 TAF，全球每年每天有约 3000 万病人服用核苷类抗艾滋病药。最重要、应用最广泛的乙肝药包括 TAF，TDF，Entecuvir 都是核苷类药物。丙肝药中的索非布韦更是丙

肝治愈中的革命性的药物，使丙肝的治愈率达到 95%以上。可见，核苷类药物在抗病毒治疗中有着不可替代的地位。

核苷类抗病毒药物有抗病毒疗效高，耐药屏障高的特点。2019-nCoV 冠状病毒是新型 RNA 病毒，有几个核苷被报道显示体外抗冠状病毒效果，如 6-azauridine，carbavir, 尤其是 Remdesivir，体外显示较强的抗冠状病毒的作用。这些核苷药物都在早期研发阶段，6-azauridine 的毒性较大，选择性较低，Carbovir 的抗病毒活性较低。Remdesivir 的临床安全性还有待探索。目前，新型冠状病毒（2019-nCoV）感染在全球，尤其在中国的扩展迅速，成为人类健康的重大威胁。因此，寻找新型核苷类抗冠状病毒药物十分必要。

I 类新药阿兹夫定（Azvudine，FNC）是由新型核苷类逆转录酶抑制剂，据 Roche（罗氏）报道，体外试验证明，阿兹夫定能显著地抑制 RNA 病毒，如丙肝病毒（IC50=24nM, JBC, 2008, 283, 2167）、手足口病毒（RNA）、非洲猪瘟病毒（RNA）及乙肝病毒（DNA 病毒 EJMC 2015,101,103），显著抑制 HIV（RNA 病毒），并且已经完成抗 HIV 二期临床试验（GQ-FNC-201），人体有效性和安全性均良好。以上数据证明阿兹夫定具有显著而广谱的抑制 RNA 和 DNA 病毒的作用，并且临床安全性良好。因此，阿兹夫定很可能具有抗冠状病毒（2019-nCoV）的作用。面对这样 2019-nCoV 病毒引起的肺炎疫情严峻的问题，研制安全有效的抗 2019-nCoV 药物乃当务之急，建议开展临床试验探索阿兹夫定抑制冠状病毒的疗效。

二、 您将服用什么药物？

本品为阿兹夫定片（FNC），英文名 Azvudine；商品名：阿兹夫定片，制剂规格：1mg/片。1 类新药阿兹夫定片是由河南师范大学常俊标校长发明，由河南真实生物科技有限公司拥有自主知识产权的新型核苷类逆转录酶抑制剂。

三、 本试验的目的是什么？

研究目的：在临床常规治疗的基础上，评价阿兹夫定片治疗新型冠状病毒感染肺炎的有效性和安全性。

四、 哪些人可以参与本试验？

1、入选标准

您应全部符合以下标准才能进入本试验：

（1） 年龄≥18 周岁（含临界值），性别不限；

（2） 呼吸道标本或血液标本实时荧光 RT-PCR 检测 2019-nCoV 核酸阳性，或者，呼吸道标本或血液标本病毒基因测序，与已知的 2019-nCoV 高度同源；

（3） 确诊为新型冠状病毒感染者，患者符合世界卫生组织（WHO）在 2020 年 1 月 28 日发布的《最新版针对新型冠状病毒的临床指南》和中国卫健委《关于印发新型冠状病毒感染的肺炎诊疗方案（试行第五版）》的诊断标准；

（4） 已签署知情同意书。

2、排除标准

您符合以下任何一项标准将不得进入本试验：

（1） 已知或怀疑对阿兹夫定片的组成成分过敏；

（2） 合并有吸收障碍综合征，或其它任何对胃肠道吸收产生影响的状况，需要静脉营养或不能口服药物的患者；

（3） 正在接受抗 HIV 病毒治疗的患者；

（4） 患者出现以下情况之一者：出现呼吸衰竭，且需要机械通气；出现休克；合并其他器官功能衰竭需 ICU 监护治疗；

（5） 妊娠期、哺乳期妇女或在试验期间及结束 6 个月内有生育计划；

（6） 给药前 12 周内参加过其它临床试验或者正在使用试验性药物；

（7） 根据研究者的判断，具有不适合参加本试验的其它情况。

五、 本试验有哪些流程及内容？

本试验共纳入 20 例符合入组条件的受试者，按 1:1 比例随机分配到试验组或对照组，每组 10 例。试验组为服用阿兹夫定片及常规治疗，对照组为常规治疗。在这期间需要您接受抽血、化验等相关检查。

治疗方案：

试验组：阿兹夫定片+常规治疗

对照组：常规治疗

阿兹夫定片：5 片/次，1 次/日，口服。

常规治疗：参照中国卫健委《关于印发新型冠状病毒感染的肺炎诊疗方案（试行第五版）》，给予受试者相应的治疗措施。

疗程：每日给药直至受试者解除隔离出院或根据病情转至相应科室治疗其他疾病或病死。

本试验为开放研究，意味着您在给药前将知道您的试验组别。

六、 本试验您需要参与多长时间？

直至受试者解除隔离出院或根据病情转至相应科室治疗其他疾病或病死。

七、 本试验您的义务是什么？

您应当了解作为这项研究的参与者，您有一些相应的义务。您需要按时到医院进行随访，接受检查。同时，您有责任向您的负责医生报告在试验过程中您身体和精神方面的任何改变，无论您认为这种改变是否与这项研究有关。

开始研究前请您务必告知我们您目前正在服用和在研究期间将要服用的任何其他药物，包括任何处方药物、非常规维生素和中草药等的非处方药物。在研究期间如果您因为任何疾病需要到医院就诊或住院治疗，请告知医生您正在参加此项试验，以便医生更好地了解您的情况。一旦发生上述情况，请尽快通知您的负责医生。

试验期间还应遵循以下合并治疗要求：

禁止使用的治疗：

试验期间，您不得使用其它抗 HIV 病毒药物，以及研究者禁止使用的其他研究药物。

允许使用的治疗：

抗病毒治疗过程中，肝损害是常见的不良反应。试验期间，如果发生肝损害，需要及时给与保肝治疗。

八、 参加本项研究可能的受益

您和社会将可能从本项研究中受益。但我们不能保证肯定受益，需获得具体研究结果数据，且经过科学统计分析后才能具体判断。您的获益可能表现为您的疾病在本试验得到有效

治疗；社会的获益可能表现为本项研究帮助开发出一种新的治疗方法，以用于患有相似病情的其他病人。

九、 研究治疗的花费是多少？

试验期间您的试验用药物及根据方案要求的所有检查均由医院为您免费提供。

十、 您可以获得更多的信息

在试验期间如果出现有关该研究药物的新信息，则您有知情权，并可以决定是否继续参与研究，如继续，则需要重新签订一份新的知情同意书。

十一、 参加本项研究可能的风险及保护措施

任何药物都有可能给您带来不适。

I 期临床试验中共入组 40 例未接受治疗的 HIV 感染者进行单次给药，未出现严重不良反应，不良反应主要为发热、头痛、头晕、恶心、呕吐、腹泻，严重程度均为 1 级，除了 2 例受试者发热采用扑热息痛（1 片/1 次，2 次，1 天）和泰诺（1 片）治疗外，均未采取任何措施自行恢复；共入组 16 例未接受治疗的 HIV 感染者进行多次给药，2mg 和 4mg 分别有 3 例和 2 例受试者发生 5 次和 2 次 AE，仅 4mg 组 1 例 1 次 AE 判定为与研究药物可能有关，严重程度Ⅱ级，表现为给药第 4 天发生中性粒细胞绝对值降低，未给与治疗，出组检查时已恢复消失/复常，其余 6 次 AE 严重程度均为 1 级，5 次 AE 判定为与研究药物可能无关，1 次 AE 肯定有关。

Ⅱ期临床试验共计入组 172 例受试者，其中 2 例受试者撤回知情同意书，15 例受试者因耐药、依从性不佳、自愿更换为自费药物、不良事件等原因退出研究，155 例完成 48 周访视。总体来说，受试者耐受性良好，对治疗方案的依从性高，发生的不良事件大部分为轻度或中度。服用本研究药物过程中发生率最高的不良反应为头晕，其次为丙氨酸氨基转移酶升高、血尿酸升高、γ-谷氨酰转移酶升高、天门冬氨酸氨基转移酶升高。

针对以上可能的风险，在参加本研究期间我们将为您安排体检，这些检查包括：生命体征（体温、心率、呼吸、血压）、指脉氧、动脉血气分析、病原核酸检测、血常规、血生化、血沉、C-反应蛋白、心肌标志物、降钙素原、血乳酸、心肌酶谱、凝血功能、传染五项、免

疫五项、呼吸道病毒九项、快速呼吸道病毒三联、T 淋巴细胞亚群检及心电图、肺部 DR/CT 等。

除此之外，您应当了解，研究中可能出现一些不可预知的不良反应，或者在研究中也可能出现一些与试验药物无关但会对您健康造成不良影响的情况，这是目前我们无法预知的。

十二、 妊娠和哺乳：

本品对妊娠或者哺乳的风险尚不明确。如果您已怀孕或正处于哺乳期，您不能参加本试验。如果您在研究期间受孕，将此信息告知您的医生是非常重要的。在入选本试验前应确保您没有怀孕。在本试验期间，您必须使用有效的避孕措施，适当的避孕方法包括宫内节育器、使用带杀精子剂的子宫帽或避孕套等非药物避孕。如您是男性，建议您在参加本试验期间采取有效的避孕措施，确保在研究期间伴侣不受孕。

试验期间，如果您的避孕措施出现任何差错，请立即通知您的负责医生，由您的负责医生和产科医生共同做出判定，您的负责医生将跟踪您/您伴侣的妊娠状态直至结果明确，包括流产或自动终止妊娠，分娩细节，是否存在任何出生缺陷，或先天畸形，或母亲的和新生儿的并发症。

十三、 研究相关损害的赔偿

如果您在研究过程中出现与参加试验相关的不适或不良医学事件，您的负责医生将采取相应的治疗措施，直到不良事件消失或恢复至参加试验前水平。

十四、 您可以自愿选择参加研究和中途退出研究

如果您选择参加本项研究，我们希望您能够坚持完成全部研究过程。

如果您在研究过程中的任何时间退出本试验，这都不会影响您和医生的关系，也不会影响您的医疗或有其他方面利益的损失，您的负责医生将安排您继续接受常规的医疗护理。

十五、 您个人信息的保密

如果您签署知情同意书同意参加此项试验，意味着您同意申办者的指定人员或代表、国家药品监督管理局及伦理委员会可以直接查阅您的病历记录。查阅的目的是确保准确收集试验资料，保证试验严格按照研究方案进行。上述人员将确保您的个人资料保密，除非在极少

数情况下，根据法律或司法程序必须向其他人员透露。试验的相关资料将有可能被用于科学研究或文章发表，但不会包括您的名字、身份、电话号码等私人信息。

除本试验以外，有可能在今后的其他研究中会再次利用您的医疗记录，同样不会披露您的个人身份。

十六、 如果您有问题或困难该与谁联系？

如果您在试验过程中发生任何与权益相关问题，可联系伦理委员会，联系人：任志刚，联系电话：18703636245。

现在该做什么

是否参加本项研究由您自己决定。您可以和您家人或者朋友讨论后再做出决定。

在您做出参加研究的决定前，请尽可能向您的医生询问有关问题，直至您对本项研究完全理解。

感谢您阅读以上材料。如果您决定参加本项研究，请您在本知情同意书上签名。您将得到一份已签署知情同意书的副本，请妥善保存。

知情同意书·同意签字页

研究题目：阿兹夫定片治疗新型冠状病毒感染肺炎的随机、开放、对照临床试验

申请单位：光山县人民医院

患者同意声明

我已经阅读和理解了所有提供给我的参加此项临床试验的相关信息，并且有时间和机会提出问题。所有我提出的问题都已经得到了满意的答复。我自愿参加此项研究。我保证我所提供的所有个人资料，包括我的病史，都是真实和尽可能准确的。我同意本研究使用我的医疗资料。

我知道我可以获得这份已签署的知情同意书的副本。

最后，我决定同意参加本项研究，并保证尽量遵从医嘱。

受试者（正楷）：

受试者签名：

联系电话：

日期： 年 月 日 时 分

医生声明

我确认已向患者解释了本试验的详细情况，包括其权利以及可能的受益和风险，并给其一份签署过的知情同意书副本。

研究者（正楷）：

研究者签名：

联系电话：

日期： 年 月 日 时 分

阿兹夫定片治疗新型冠状病毒感染肺炎的随机、开放、对照临床试验受试者随机表

The random table that was generated by applying SAS at 1:1

1. 试验项目信息

方案编号	FNC-Hope1	
随机软件	SAS 9.4	
随机日期	2020.02.15	
受试者随机参数	种子数	456
	随机方法	完全随机
	样本量	20
	编号范围	01~20

2. 受试者随机分组表

随机编号	试验组别	随机编号	试验组别
01	A	11	A
02	A	12	B
03	A	13	B
04	A	14	B
05	A	15	B
06	B	16	A
07	B	17	B
08	B	18	B
09	B	19	A
10	A	20	A

备注：A 试验组 B 对照组

3. 随机信息拆阅记录

本随机表拆阅人： 拆阅日期：
